# Double agents: genes with both oncogenic and tumor-suppressor functions

**DOI:** 10.1038/s41389-018-0034-x

**Published:** 2018-03-13

**Authors:** Libing Shen, Qili Shi, Wenyuan Wang

**Affiliations:** 1 Interdisciplinary Research Center on Biology and Chemistry, Shanghai Institute of Organic Chemistry, Chinese Academy of Sciences, Shanghai, 200032 China; 2 Department of Rehabilitation Medicine, Hua Shan Hospital, Fudan University, Shanghai, 200040 China

## Abstract

The role of genetic components in cancer development is an area of interest for cancer biologists in general. Intriguingly, some genes have both oncogenic and tumor-suppressor functions. In this study, we systematically identified these genes through database search and text mining. We find that most of them are transcription factors or kinases and exhibit dual biological functions, e.g., that they both positively and negatively regulate transcription in cells. Some cancer types such as leukemia are over-represented by them, whereas some common cancer types such as lung cancer are under-represented by them. Across 12 major cancer types, while their genomic mutation patterns are similar to that of oncogenes, their expression patterns are more similar to that of tumor-suppressor genes. Their expression profile in six human organs propose that they mainly function as tumor suppressor in normal tissue. Our network analyses further show they have higher network degrees than both oncogenes and tumor-suppressor genes and thus tend to be the hub genes in the protein–protein interaction network. Our mutation, expression spectrum, and network analyses might help explain why some cancer types are specifically associated with them. Finally, our results suggest that the functionally altering mutations in “double-agent” genes and oncogenes are the main driving force in cancer development, because non-silent mutations are biasedly distributed toward these two gene sets across all 12 major cancer types.

## Introduction

Cancer is a series of diseases featured with abnormal cell growth and the potential of spreading to the other body parts. Many biologists share the view that cancer is an evolutionary legacy^1^. Thus, besides environmental factors, the genetic factors shaped by evolution play a crucial role in cancer development. The cells of the multicellular organisms harbor both oncogenes and tumor-suppressor genes. The former could cause normal cell to grow out of control and become cancer, while the latter protects them from degenerating into cancer cells. They appear to be two antithetical gene categories in oncogenesis. However, paradoxically, some genes exhibit both oncogenic and tumor-suppressor functions^2–5^. For example, NOTCH receptors, the vital components of the evolutionarily conserved Notch signaling pathway, can be classified as both oncogene and tumor-suppressor gene^6^. It plays an oncogenic role in T-lineage acute lymphoblastic leukemia while it performs tumor-suppressor function in squamous epithelial cells^7,8^. These results indicate that a gene could have dual roles in oncogenesis under different cellular contexts.

Conceptually, cancer is a result of consecutive somatic mutation accumulation^9–11^. Many studies show that both the gain of function in oncogenes and the loss of function in tumor-suppressor genes are required for the development of cancer from a normal cell^12–16^. For a diploid organism, gain-of-function mutations are often dominant or semi-dominant, whereas loss-of-function mutations are usually recessive. Two-hit hypothesis of oncogenesis proposes that the development of cancer is initiated by the loss of both alleles of a tumor-suppressor gene^17^. Retinoblastoma is an example of two-hit cancers, in which both *RB1* genes, a tumor-suppressor protein, are inactivated^18^. For a gene with both oncogenic and tumor-suppressor potentials, it is possible that one single mutation event would unleash its oncogenic power and abolish its tumor-suppressor function. Theoretically, one such mutation event would be enough to trigger the carcinogenic cascade in normal cells. Thus, it is an important and interesting question to ask whether some cancers need less mutation steps to develop than the others and what kind of genes play driver roles in the development of these “one-hit” cancers.

Given the special role of these genes in oncogenesis, it is important for cancer biologists to learn how many of them exist and what are their functions in order to gain a better understanding of their contribution to oncogenesis. Here we present a study focusing on the genes with both oncogenic and tumor-suppressor functions. Based on the available databases, we identified these “double-agent” genes through text mining. We found that the proportions of cancer types associated with these genes were significantly different from global cancer statistics and most of them were transcription factors or kinases. We detailedly studied them as a gene set, which exhibit duality in their biological functions. We also used the mutation and expression data from The Cancer Genome Atlas (TCGA) project to compare their mutation and expression pattern with oncogenes and tumor-suppressor genes. Our results showed that, in 12 major cancer types, their mutation patterns resembled those of oncogenes, while their expression patterns were more similar to those of tumor-suppressor genes. We further used the interactome data to study their network properties and roles in protein–protein interaction (PPI) network and found that they tended to be the hub genes within the network. Hopefully, our study can provide cancer biologists with the knowledge of these genes from various perspectives.

## Materials and methods

### Gene set and cancer information gathering

Oncogenes (ONCs) were downloaded from Network of Cancer Genes database (NCG 5.0)^19^. Tumor-suppressor genes (TSGs) were downloaded from Tumor Suppressor Gene database (TSGene 2.0)^20^. The genes overlapped between two databases were viewed as the candidates for “double-agent” genes. These candidate genes were searched in the GeneRIF database (ftp://ftp.ncbi.nih.gov/gene/GeneRIF/) which provide the literature annotations for our candidates. Then we manually curated these candidate genes according to literature evidence. Only if literature evidence reported one candidate gene as both oncogene and tumor-suppressor gene, it would be identified as “double-agent” genes (please see supplemental information for the more detailed workflow of our database search and text mining process). Literature evidence shows that “double-agent” genes are actually proto-oncogenes with tumor-suppressor function. Thus, we abbreviatively named them as POTSF genes in this study.

The cancer type information for each POTSF gene was extracted from its literature evidence. We further gleaned the specific cancer information for each POTSF gene from Cancer Genetics Web (http://www.cancerindex.org/geneweb/), which is also based on literatures and used as a complement to our literature mining result.

### Functional enrichment analysis and gene expression information gathering

In order to investigate their biological functions, we used DAVID (the Database for Annotation, Visualization and Integrated Discovery) Bioinformatics Resources to perform the gene-GO term enrichment analyses for POTSF genes, oncogenes (ONCs), and tumor-suppressor genes (TSGs)^21^. To visualize the Gene Ontology (GO) results, we used ggplot2 package for GO result display.

The expression information for each POTSF gene in five tissues was retrieved from Genecard database^22^. Genecard database has the tissue expression values (microarray and RNA-Seq data) for an inquiry gene. We gleaned the expression values from top five tissues for each POTSF gene and organized these tissue types into five major categories: internal and musoskeletal tissue, blood and immune tissue, secretory tissue, nervous tissue, and reproductive tissue (please see supplemental data for each POTSF gene's specific expression information). Genecard database shows that some POTSF genes are ubiquitously expressed and some of them have no expression information.

We used the R package Venn Diagram to display POTSF gene expression information in a Venn diagram format^23^.

### Somatic mutation data for 12 major cancer types

In order to explore the mutation patterns of POTSFs, ONCs, TSGs, and non-cancer related genes (NCRGs) in cancers, we downloaded the somatic mutation data from one published TCGA project^24^. The data include 3281 cancer cases from 12 major cancer types with a total of 617,354 somatic mutations in 20,947 genes. The 12 major cancer types are bladder urothelial carcinoma (BLCA), breast adenocarcinoma (BRCA), colon and rectal carcinoma (COADREAD), glioblastoma multiforme (GBM), head and neck squamous cell carcinoma (HNSC), kidney renal clear cell carcinoma (KIRC), acute myeloid leukemia (LAML), lung adenocarcinoma (LUAD), lung squamous cell carcinoma (LUSC), ovarian serous carcinoma (OV), and uterine corpus endometrial carcinoma (UCEC).

In 617,354 somatic mutations, there are 39,869 nonsense mutations, 134,635 silent mutations, 916 in-frame insertions, 5134 frameshift insertions, 10,743 frameshift deletions, 3590 in-frame deletions, 10,190 splice site mutations, 784 nonstop mutations, 416,847 missense mutations, and 10,659 non-coding RNA mutations. We filtered 134,635 silent mutations and 10,659 non-coding RNA mutations from a total of 617,354 somatic mutations, because these mutations will not alter the amino acid sequence of a protein. We only investigated the mutation patterns of 472,060 non-silent mutations in this study.

The mutation rate for each gene was calculated as follows. First, we calculated the total number of mutations (including both non-silent and silent mutations) for each gene in each cancer type. Second, the total number of mutations for each gene was divided by the number of cases in each cancer type. Third, the average number of mutations for each gene in each cancer type was divided by the gene length and the quotient is the mutation rate for each gene in each cancer type.

### RNA-Seq data for 12 major cancer types and 6 normal human organs

In order to explore the expression patterns of POTSFs, ONCs, TSGs, and NCRGs in cancers, we downloaded the RNA-Seq data of 12 major cancer types from the cBioPortal, a website hosting the data of TCGA project^25^. In the downloaded RNA-Seq data, there are 129 samples of BLCA, 817 samples of BRCA, 382 samples of COADREAD, 166 samples of GBM, 279 samples of HNSC, 469 samples of KIRC, 173 samples of LAML, 230 samples of LUAD, 501 samples of LUSC, 307 samples of OV, and 333 samples of UCEC, respectively.

TCGA project uses the RSEM (RNA-Seq by Expectation-Maximization) value to quantify each gene’s expression level in cancer samples. To make different gene expression levels from different samples comparable within a cancer type, we used the normalized RSEM values to describe the expression value for each gene. In this study, the RSEM values are normalized to medians instead of means. Due to the multiple samples in each cancer type, we calculated the average RSEM value for each gene based on the RSEM values from multiple cancer samples.

The RNA-Seq data of six human organs (brain, cerebellum, heart, kidney, liver, and testis) were downloaded from the supplementary information of Brawand et al.^26^. We calculated the RPKM (Reads Per Kilobase per Million mapped reads) value for each gene based on the downloaded data (unique read coverage per exon). Due to the uneven number of samples in some organs, we used the mean RPKM value if multiple RPKM values were available for each human organ. We transformed the RPKM values into the log_2_(RPKM) values and then calculated the *Z*-score for every log_2_(RPKM) value within each organ in order to render the gene expression values comparable among different organs.

### Network analysis

We downloaded the interactome data from mentha database (http://mentha.uniroma2.it/) and used them to perform the network analysis for POTSFs, ONCs, TSGs, and NCRGs^27^. For each gene with PPI information, we calculated its degree *k* and clustering coefficient *C*(*k*) (please see supplemental information for the more detailed description of our network analysis).

We used Cytoscape 3.4.0 to generate the undirected PPI network for POTSFs and their interaction proteins^28^. MCODE, a Cytoscape plugin, was used to extract the highly interconnected regions in this network with default parameters^29^.

### Statistical analysis

The R package (version 3.2.4) was used to perform statistical analyses and *P*-value smaller than 0.05 was viewed as statistically significant in this study. Chi-square test was employed to compare the percentages of top 10 cancer types associated with POTSFs and the percentages of these cancer types in 2012 global cancer statistics^30^. Two-sided Kolmogorov–Smirnov test was employed to compare the mutation patterns of 472,060 non-silent mutations from POTSFs, ONCs, TSGs, and NCRGs in 12 cancer types. Wilcoxon test was employed to compare the expression levels of POTSFs, ONCs, TSGs, and NCRGs in 12 cancer types and in 6 normal human organs.

## Results

### Number, classification, and biological function of proto-oncogenes with tumor-suppressor function

Through database search and literature annotation, we identified 83 POTSF genes, 1320 ONCs, and 952 TSGs in this study. According to literature annotation, we find that many POTSF genes are either transcription factors or kinases. Thus, we classified POTSF genes into three categories: transcription factors, kinases, and others (Table 1). About half of POTSF genes are transcription factors (41/83) and about one-sixth of them are kinases (13/83). Compared with POTSF genes, the percentage of transcription factors and kinases are much lower in ONCs and TSGs (Figure S2a, S2c and S2e).

**Table 1:** The classification of proto-oncogenes with tumor-suppressor function (POTSF)

Classification	POTSF genes
Transcription factors	*BRCA1, CAMTA1, CBFA2T3, CDX2, CREB3L1, CREBBP, DDB2, DNMT1, DNMT3A, ETV6, EZH2, FOXA1, FOXL2, FOXO1, FOXO3, FOXO4, FOXP1, FUS, IRF4, KLF4, KLF5, NCOA4, NOTCH1, NOTCH2, NOTCH3, NPM1, NR4A3, PAX5, PML, PPARG, RB1, RUNX1, SMAD4, STAT3, TCF3, TCF7L2, TP53, TP63, TRIM24, WT, ZBTB16*
Kinases	*BCR, CHEK2, EPHA1, EPHA3, EPHB4, FLT3, MAP2K4, MAP3K4, MST1R, NTRK3, PRKAR1A, PRKCB, SYK*
Others	*ARHGEF12, BCL10, BRCA2, CBL, CDC73, CDH11, CDKN1B, DCC, DDX3 × , DICER1, FAS, FAT1, GPC3, IDH1, IKZF2, LIFR, NF2, NUP98, PHF6, PTPN1, PTPN11, RHOA, RHOB, SH2B3, SLC9A3R1, SOCS1, SPOP, SUZ12, WHSC1L1*

**Fig. 1:**
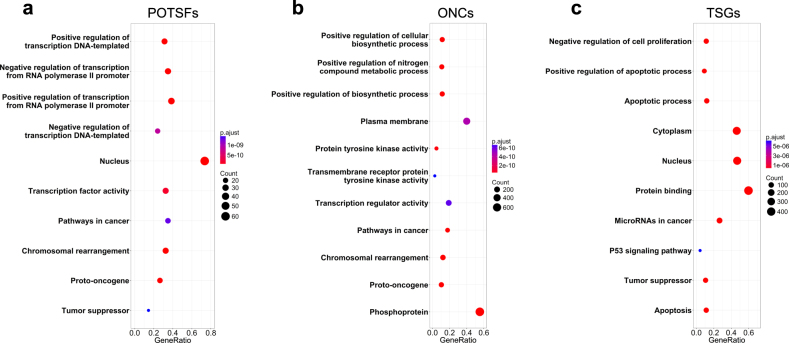
GO term enrichment analyses of proto-oncogenes with tumor-suppressor function (POTSFs), oncogenes (ONCs), and tumor-suppressor genes (TSGs). **a** GO term enrichment results for POTSFs. **b** GO term enrichment results for ONCs. **c** GO term enrichment results for TSGs

In order to investigate their functions as a gene set, we performed the gene-GO term enrichment analyses for POTSFs, ONCs, and TSGs. The DAVID GO term results are shown in Fig. 1. For POTSFs, the DAVID results intriguingly show that their biological process exhibits a contradicting regulation of transcription—they both positively and negatively regulate transcription (Fig. 1a). GO keywords clearly show that they are both proto-oncogene and tumor suppressors. For ONCs, the DAVID results show that they mostly involve positive regulation of a variety of biological processes and GO keywords identify them as proto-oncogenes (Fig. 1b). For TSGs, the DAVID results show that they mainly participate in the inhibition of cell proliferation and the promotion of cell apoptosis (Fig. 1c). GO keywords identify them as tumor suppressors. The KEGG (Kyoto Encyclopedia of Genes and Genomes) pathways and PIR (Protein Information Resource) keywords of POTSFs are partly overlapped with ONCs, while their cellular components and molecular functions are partly or completely overlapped with TSGs. Interestingly, ONCs is the only gene set that positively regulates nitrogen and metabolic process among three gene categories.

### Cancer types associated with POTSFs and the expression spectrum of POTSFs

**Fig. 2:**
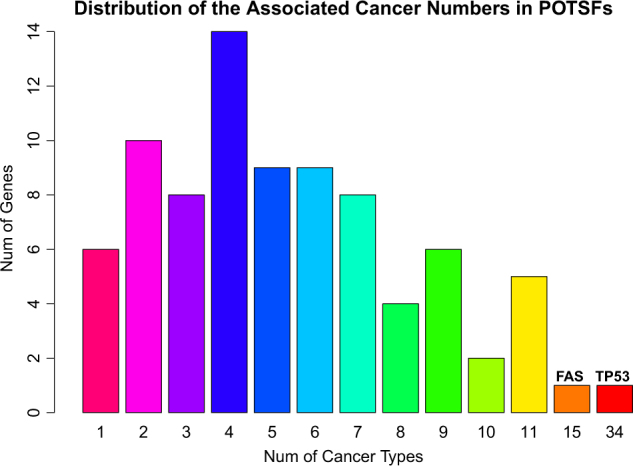
The distribution of the associated cancer type numbers in POSTFs. *TP53* is associated with 34 different cancer types and *FAS* is associated with 15 different cancer types

Based on database and literature searches, we also identified the cancer types specifically associated with each “double-agent” gene (supplemental data). The majority of them are associated with at least two types of cancer (Fig. 2). *TP53* and *FAS* are top two POTSF genes in terms of the number of associated cancer types, which are associated with 34 and 15 cancer types, respectively. The number of associated cancer types shows that most POTSF genes are not disease specific.

**Fig. 3:**
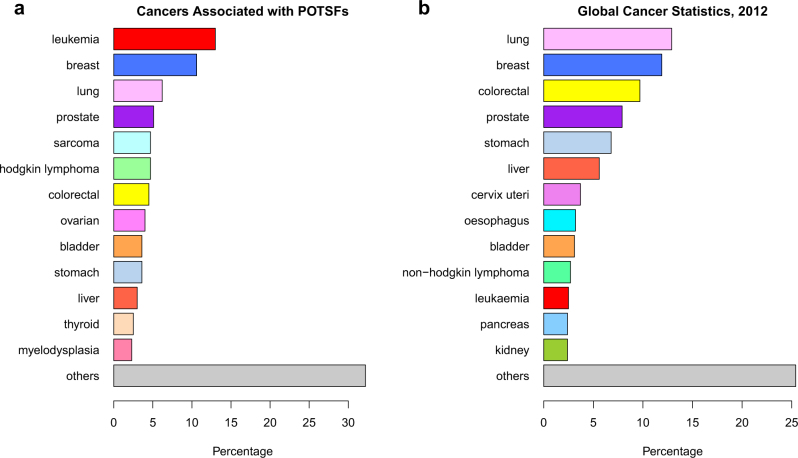
The cancer type information for POTSFs and from 2012's global cancer statisitics. **a** The percentages of the cancer types associated with POTSFs.** b** The percentages of the cancer types reported in 2012’s global cancer statistics. The same cancer type is marked with the same color in (**a**) and (**b**); e.g., leukemia is marked with red color in both (**a**) and (**b**)

We further investigated what types of cancer were specifically associated with POTSF genes. Our result shows that leukemia, breast cancer, and lung cancer are three most common cancer types associated with POTSF genes (Fig. 3a). We also compared our result with 2012 global cancer statistics (Fig. 3b). Chi-square test shows that there is a detectable statistical difference between the percentages of top 10 cancer types associated with POTSFs and 2012 global cancer statistics (*P*-value = 0.01674, leukemia: 13 vs. 2.5%, breast: 10.6 vs. 11.9%, lung: 6.2 vs. 12.9%, prostate: 5.1 vs. 7.9%, sarcoma: 4.7 vs. 1%, Hodgkin lymphoma: 4.7 vs. 0.5%, colorectal: 4.5 vs. 9.7%, ovarian: 4 vs. 1.3%, bladder: 3.6 vs. 3.1%, and stomach: 3.6 vs. 6.8%). Leukemia, Hodgkin lymphoma, sarcoma, and ovarian cancer are over-represented by “double-agent” genes, while more common cancers such as lung, prostate, colorectal, and stomach cancers are under-represented.

**Fig. 4:**
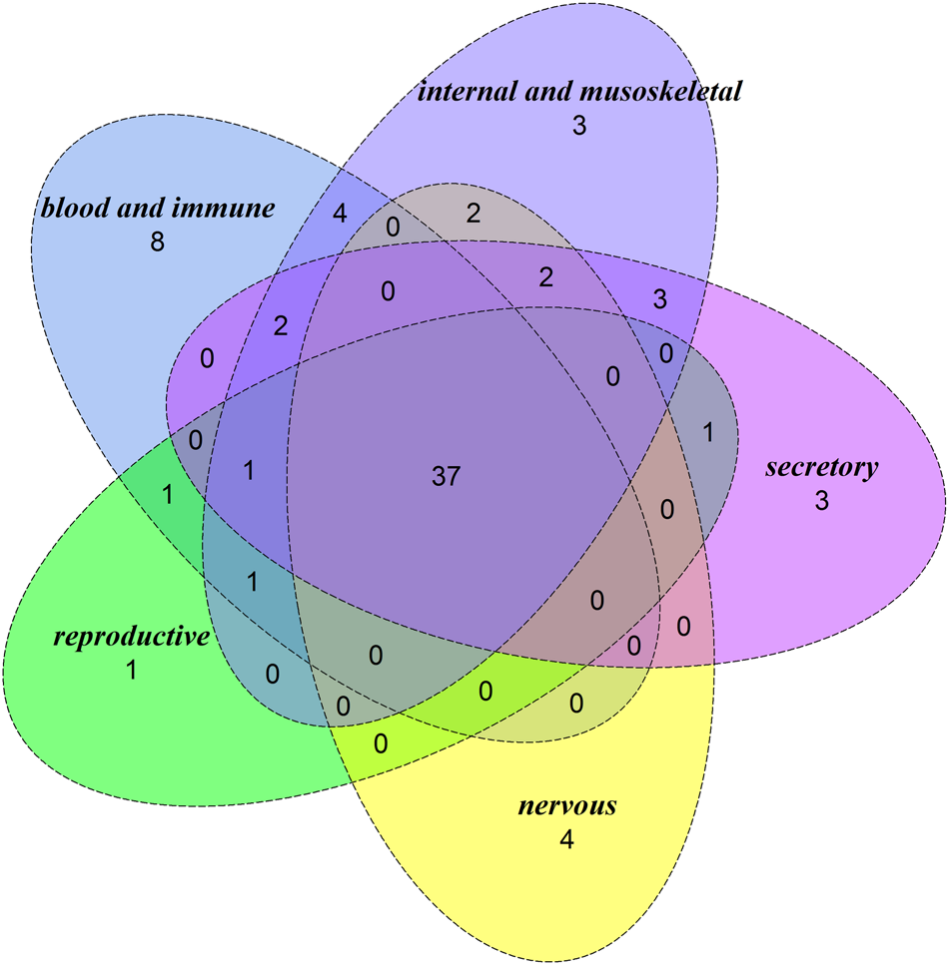
Expression spectrum of POTSFs in five tissues. Internal and musoskeletal tissue includes lung, kidney, liver, colon, heart, muscle, etc. Blood and immune tissue includes thymus, bone marrow, peripheral blood, lymph node, etc. Secretory tissue includes skin, prostate, placenta, pancreas, etc. Nervous tissue includes forebrain, retina, cerebellum, etc. Reproductive tissue includes testis and ovary. Please see supplemental data for each POTSF-specific expression information

To investigate whether their expression spectrum might be related to their associated cancer types, we extracted the expression information for POTSFs from Genecard database. We used five major tissue types for displaying the expression spectrum of POTSFs (Fig. 4). They are blood and immune, internal and musculoskeletal, secretory, nervous, and reproductive systems. Figure 4 shows that more than one-third of POTSFs (37/83) are ubiquitously expressed in five tissue types and only 19 of them are tissue specific. Among tissue-specific genes, there are more POTSFs expressed in blood and immune system than other tissues (8 out of 19). This result is consistent with our observation that different types of leukemia are the most common cancer type associated with POTSFs.

### Distribution of non-silent mutations in POTSFs, ONCs, TSGs, and NCRGs and their mutation rates across 12 cancer types

To investigate the non-silent mutation distribution in four gene sets across different cancer types, we used the cancer somatic mutation data in 12 major cancer types. We extracted the non-silent mutation information for each gene in four gene sets if the information was available.

**Fig. 5:**
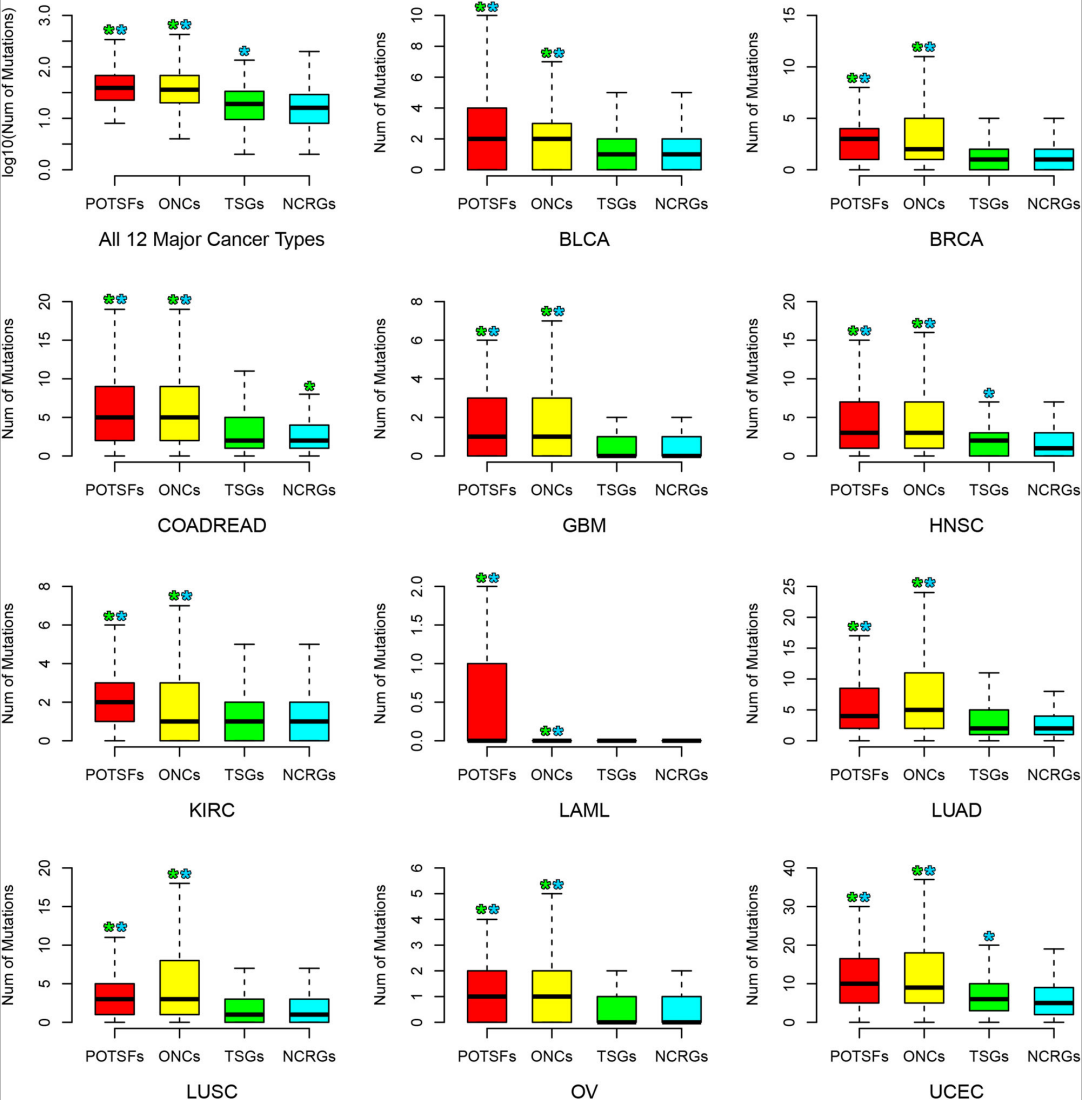
The distribution of non-silent mutations in POTSFs, ONCs, TSGs, and NCRGs across 12 cancer types. The star indicates the statistical difference (*P*-value < 0.05, Kolmogorov–Smirnov test) between two gene sets. The star is placed on the statistically higher gene set and the color of star indicates the corresponding gene set

In Fig. 5, the first panel shows the general non-silent mutation profiles of POTSFs, ONCs, TSGs, and NCRGs in all 12 major cancer types. POTSFs and ONCs averagely have 70 and 58 non-silent mutations per gene (median 39 and 36 per gene), while TSGs and NCRGs averagely have 24 and 23 non-silent mutations per gene (median 19 and 16 per gene). Kolmogorov–Smirnov tests show that POTSFs and ONCs harbor the significantly higher number of non-silent mutations than TSGs and NCRGs (*P*-values < 7 × 10^−19^, 4 × 10^−13^, 2 × 10^−16^, and 2 × 10^−16^). There is no statistical difference detected in the number of non-silent mutations between POTSFs and ONCs (*P*-values = 0.66), whereas TSGs harbor significantly more non-silent mutations than NCRGs (*P*-values = 0.00408).

Then we examined non-silent mutation patterns of four gene sets in each cancer type (Fig. 5). Interestingly, their mutation patterns show variations among different cancer types. In LAML, POTSFs averagely have the biggest number of average non-silent mutations among four gene sets (POSTFs: mean 3.3 per gene vs. ONCs: mean 0.27 per gene, TSGs: mean 0.08 per gene, NCRGs: mean 0.07 per gene, respectively), although Kolmogorov–Smirnov test shows that there is no detectable statistical difference between POSTFs and ONCs (*P*-values = 0.2034). In LUAD and LUSC, ONCs averagely have more non-silent mutations than POTSFs (mean 9.7 per gene vs. 7.9 per gene in LUAD and mean 6.7 per gene vs. 6.1 per gene in LUSC), although there is no statistical significance detected (*P*-values = 0.28 and 0.22). In COADREAD, NCRGs averagely have more non-silent mutations than TSGs (mean 3.1 per gene vs. 3.5 per gene, *P*-values = 0.000553), while in HNSC and UCEC, TSGs averagely have more non-silent mutations than NCRGs (mean 2.3 per gene vs. 2 per gene and mean 7.3 per gene vs. 6.7 per gene, *P*-values = 0.0097 and 0.0019), which are not observed in the other cancer types. In the case of COADREAD, although our result shows that NCRGs have the smaller number of non-silent mutations per gene than TSGs (mean 3.1 per gene vs. 3.5 per gene), one of NCRGs, *APC*, has 257 non-silent mutations, which significantly shifts the mutation distribution towards NCRGs in COADREAD. It is why Kolmogorov–Smirnov test shows that NCRGs have significantly more non-silent mutations than TSGs in COADREAD.

**Fig. 6:**
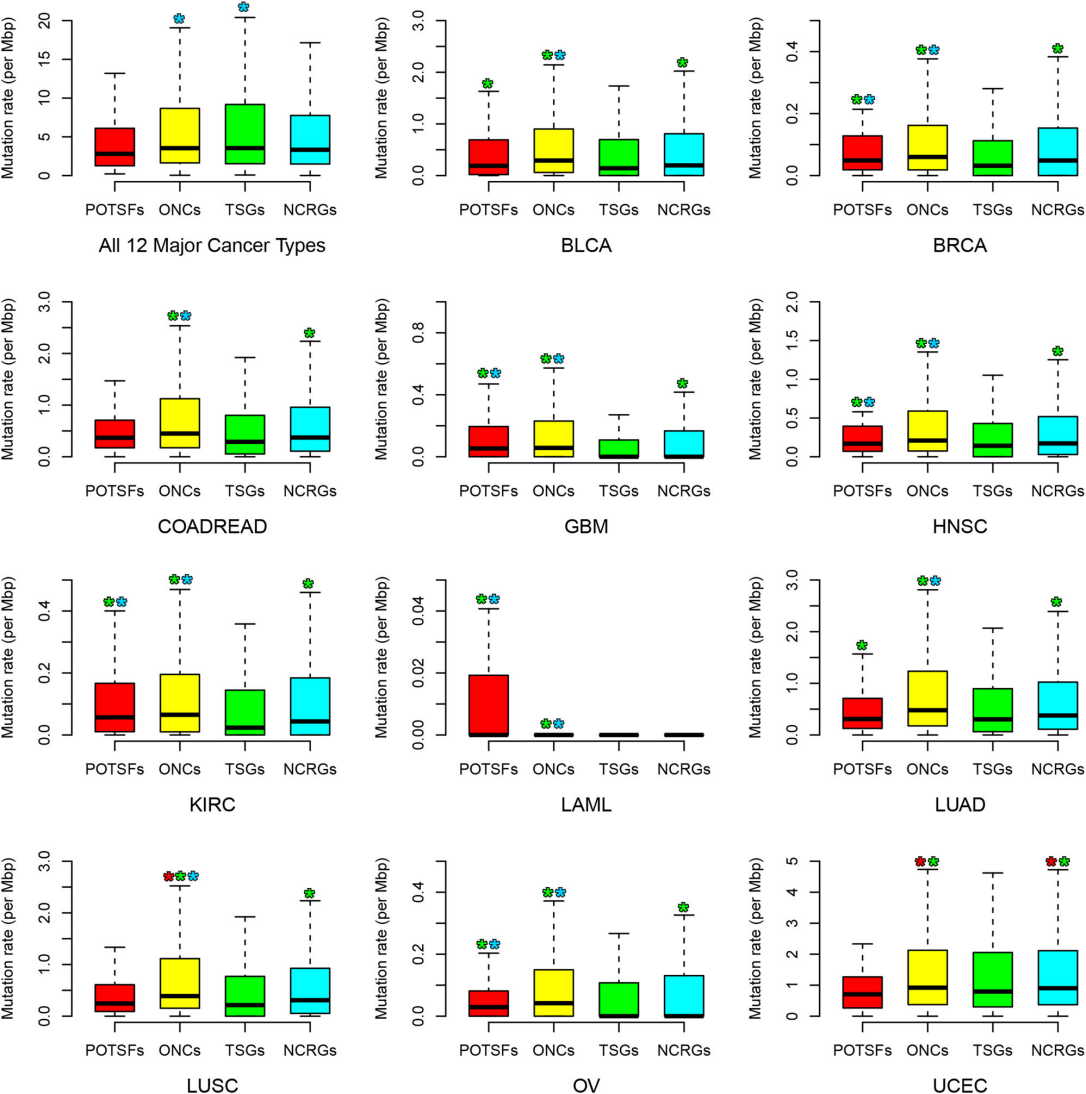
The mutation rates of POTSFs, ONCs, TSGs, and NCRGs across 12 cancer types. The star indicates the statistical difference (*P*-value < 0.05, Kolmogorov–Smirnov test) between two gene sets. The star is placed on the statistically higher gene set and the color of star indicates the corresponding gene set

The mutation rates of POTSFs, ONCs, and TSGs show a slightly different pattern from their non-silent mutation distributions across 12 cancer types (Fig. 6). First, in all 12 cancer types combined, there is no statistical difference detected among three cancer-related gene sets (POTSFs, ONCs, and TSGs) in terms of the mutation rate, although ONCs and TSGs show higher mutation rates than NCRGs (*P*-values = 0.01407, *P*-values = 0.03656, the first panel of Fig. 6). In BRCA, GBM, HNSC, KIRC, LAML, and OV, POTSFs and ONCs exhibit higher mutation rates than TSGs and NCRGs, which is very similar to their non-silent mutation distribution patterns in these six cancer types. In BLCA, COADREAD, and LUAD, ONCs exhibit higher mutation rates than TSGs and NCRGs, while POTSFs only exhibit higher mutation rates than TSGs. The mutation rates of POTSFs, ONCs, and TSGs in LUSC and UCEC are quite different from their non-silent mutation distribution patterns in these two cancer types. In LUSC, ONCs have the highest mutation rate among four gene sets and there is no mutation rate difference detected between POTSFs and TSGs or NCRGs. In UCEC, ONCs and NCRGs have higher mutation rates than POTSFs and TSGs. We notice that there is a mutation rate discrepancy between TSGs and NCRGs. TSGs have higher mutation rate than NCRGs in all 12 cancer types combined, whereas NCRGs have higher mutation rate than TSGs in each individual cancer type. We found that this discrepancy was due to too many outliers in TSGs if we calculated their mutation rates in all 12 cancer types combined. There are much less outliers in TSG gene set in each individual cancer type.

Both the non-silent mutation distributions and mutation rates in four gene sets propose that different types of cancers underwent different mutation processes and potential cancerous mutations are more prone to happen in POTSFs and/or ONCs. We also examined the distribution of potential gain-of-function mutations and potential loss-of-function mutations in POTSFs, ONCs, TSGs, NCRGs, and their non-silent and silent mutation rate across 12 cancer types in this study (Figure S4, S5, S6 and S7). Please see supplemental information for more details.

### Expression patterns of POTSFs, ONCs, TSGs, and NCRGs across 12 cancer types and 6 human normal organs

**Fig. 7:**
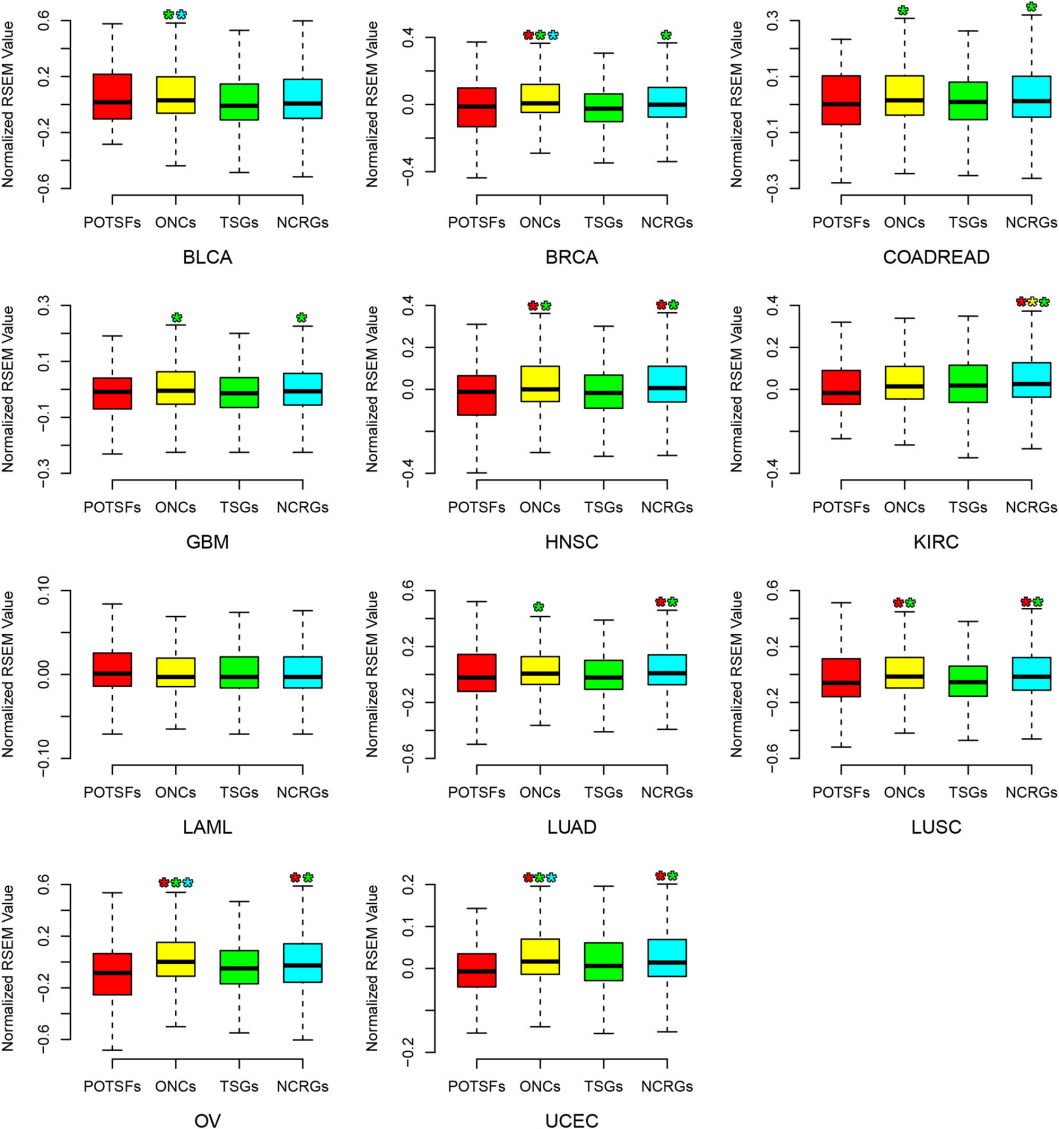
The distribution of the RNA-Seq expression values of POTSFs, ONCs, TSGs, and NCRGs across 12 cancer types. The star indicates the statistical difference (*P*-value < 0.05, Kolmogorov–Smirnov test) between two gene sets. The star is placed on the statistically higher gene set and the color of star indicates the corresponding gene set

To understand their possible roles in oncogenesis from expression perspective, we further examined the expression levels of POTSFs, ONCs, TSGs, and NCRGs across 12 cancer types. In most cancer types, ONCs averagely have a higher expression level than TSGs (Fig. 7). In BLCA, the average expression level from high to low is ONCs, POTSFs, NCRGs, and TSGs; in BRCA and GBM, the average expression level from high to low is ONCs, NCRGs, POTSFs, and TSGs; in COADREAD and LUSC, the average expression level from high to low is ONCs, NCRGs, TSGs, and POTSFs; in HNSC, the average expression level from high to low is NCRGs, ONCs, POTSFs, and TSGs; in KIRC, the average expression level from high to low is NCRGs, TSGs, ONCs, and POTSFs; in LAML, the average expression level from high to low is POTSFs, ONCs, NCRGs, and TSGs; in LUAD, the average expression level from high to low is NCRGs, ONCs, TSGs, and POTSFs; in OV and UCEC, the average expression level from high to low is ONCs, NCRGs, TSGs, and POTSFs.

**Fig. 8:**
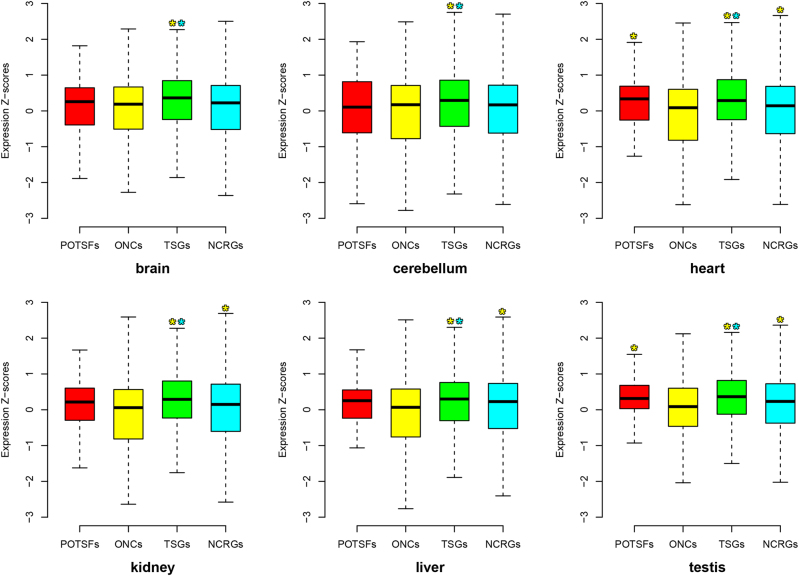
The distribution of the RNA-Seq expression values of POTSFs, ONCs, TSGs, and NCRGs in six normal human organs. The star indicates the statistical difference (*P*-value < 0.05, Kolmogorov–Smirnov test) between two gene sets. The star is placed on the statistically higher gene set and the color of star indicates the corresponding gene set

The expression patterns of POTSFs, ONCs, TSGs, and NCRGs are more diversified than their mutation patterns in 12 cancer types. Although ONCs usually have a significant higher expression level than TSGs in most cancer types, NCRGs have a significant higher expression level than the other three gene sets in KIRC and there is no statistically significant expression difference detected among four gene sets in LAML. Thus, different cancers have relatively diversified expression profiles. Such expression diversity probably attributes to the specific tissue background from where cancer cells stem. Thus, we also examined the expression patterns of POTSFs, ONCs, TSGs, and NCRGs in normal human brain, cerebellum, heart, kidney, liver, and testis. Figure 8 shows the expression patterns of POTSFs, ONCs, TSGs, and NCRGs in these six organs. There are some differences for the expression levels of POTSFs, ONCs, and NCRGs among six organs, but TSGs always have significantly higher expression levels than ONCs and NCRGs in all six organs we examined. Although our expression analysis shows that TSGs have the similar expression level as ONCs in kidney cancer (KIRC), they do exhibit a significantly higher expression level than ONCs in normal kidney. Thus, from the expression perspective, KIRC and LAML could be a consequence of the down-regulation of TSGs instead of the up-regulation of ONCs, while the other 10 cancers are a result of both the down-regulation of TSGs and the up-regulation of ONCs.

In our expression analyses, we notice one phenomenon that the expression level of POTSFs is more akin to TSGs in both 12 cancer types and 6 human organs. No statistical expression difference is detected between POTSFs and TSGs in six human organs, while the expression difference is detected between POTSFs and ONCs in BRCA, HNSC, LUSC, OV, UCEC, heart, and testis.

### Network analysis for POTSFs, ONCs, TSGs, and NCRGs

**Fig. 9:**
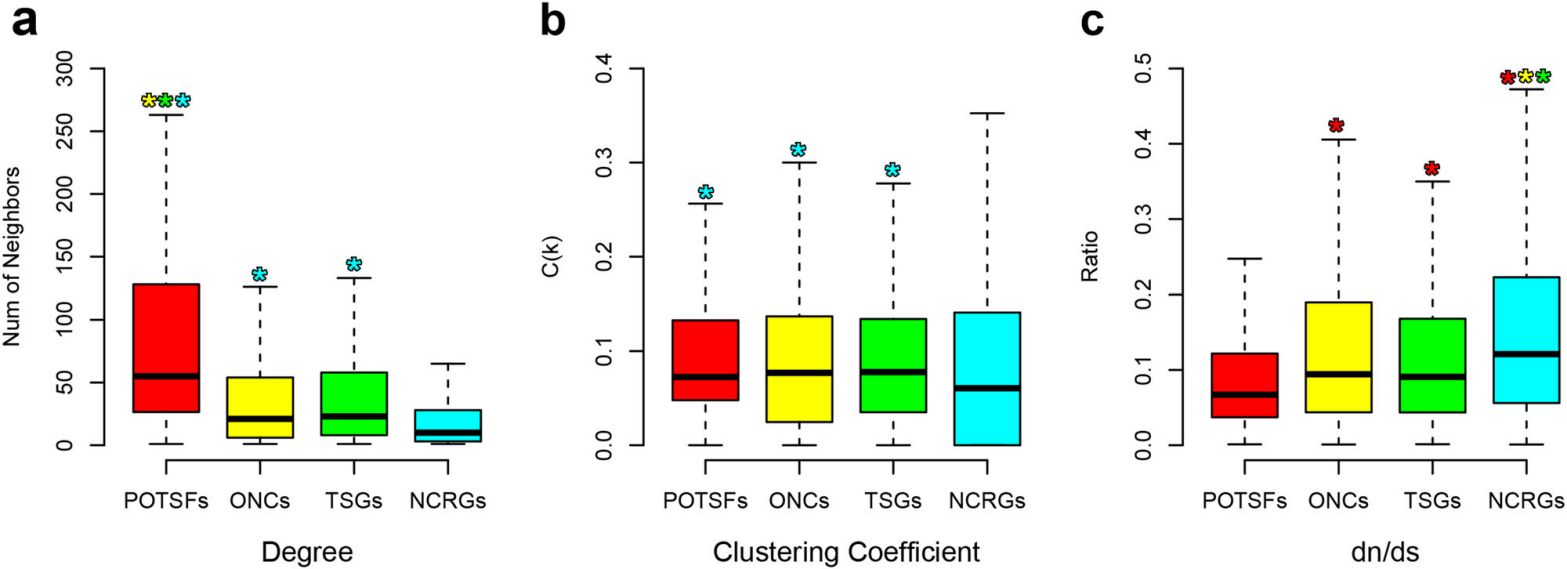
The network property and evolutionary pressure analyses for POTSFs, ONCs, TSGs, and NCRGs. **a** The network degrees of four gene sets. **b** The clustering coefficients of four gene sets. **c** The *d*_N_/*d*_S_ values of four gene sets

A coding gene usually exerts its function through interacting with other molecular entities, which is often another protein. In order to get a comprehensive view of their roles in oncogenesis, we used the PPI information to examine the network properties of POTSFs, ONCs, TSGs, and NCRGs. First, we investigated the network degree for each POTSF, ONC, TSG, or NCRG gene. Degree is a measure of the number of interacting neighbors for a node (gene). Figure 9a shows that POTSFs have the highest degree among four gene sets with significant statistical differences. Averagely, a POTSF has 104 interacting neighbors, whereas an ONC or a TSG has only 50 and a NCRG has 25. This result proposes that POTSFs are more likely to be the hub genes in PPI network. Second, we calculated the clustering coefficient for four gene sets. Clustering coefficient measures a node’s modularity in a network, i.e., the degree to which nodes in a network tend to cluster together. In PPI network, modularity often implies certain biological function. Figure 9b shows that there is no statistical difference in term of the clustering coefficients among POTSFs, ONCs and TSGs, but they have significantly higher clustering coefficients than NCRGs. It proposes that POTSFs, ONCs, and TSGs are more likely to form a biological module through PPI, while NCRGs are less likely. Third, we checked the evolutionary pressure on POTSFs, ONCs, TSGs, and NCRGs. If a gene played an important role in a biological network, it usually would have experienced greater evolutionary pressure and thus had a lower nonsynonymous to synonymous substitution ratio (*d*_N_/*d*_S_ values, please see supplemental information for how we calculated *d*_N_/*d*_S_ values for four gene sets). Figure 9c shows that POTSFs have the lowest *d*_N_/*d*_S_ values and NCRGs have the highest. The *d*_N_/*d*_S_ values of ONCs and TSGs are between that of POTSFs and NCRGs. Thus, the *d*_N_/*d*_S_ values indicate that POTSFs perform more important biological roles than the other three gene sets in PPI network.

**Fig. 10:**
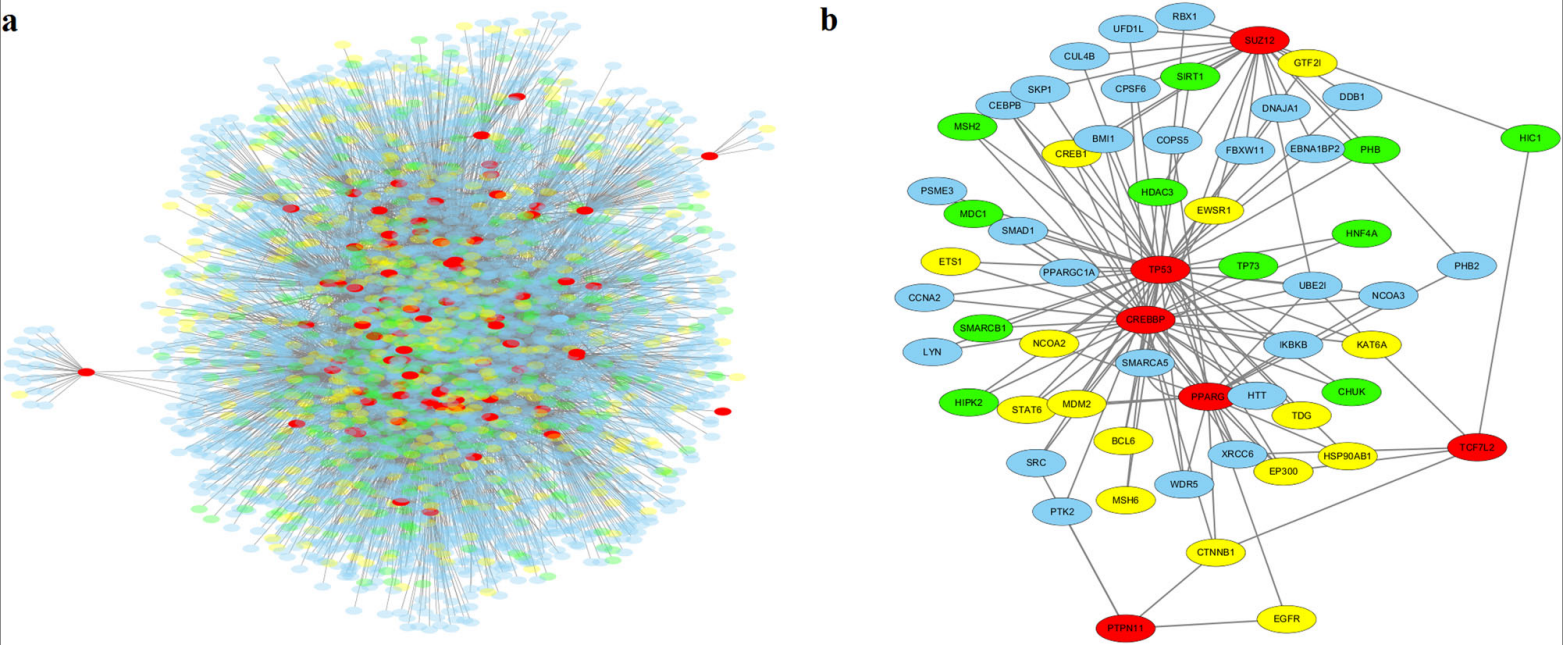
The protein-protein interaction network graph for POTSFs and its most interconnected module. **a** Protein–protein interaction network of POTSFs, ONCs, TSGs, and NCRGs. **b** The most interconnected region of the network. POTSFs are represented by red color. ONCs are represented by yellow color. TSGs are represented by green color. NCRGs are represented by blue color

For a clearer view of the network property analyses above, we generated an undirected PPI network which includes all POTSFs and their interacting proteins from the other three gene sets (Fig. 10). In this PPI network, there are a total of 4250 nodes (genes) and 8629 edges (interactions). Among the 4250 nodes, 83 of them are POTSFs, 469 are ONCs, 420 are TSGs, and 3278 are NCRGs. Figure 10a shows that most of POTSFs locate in the central region of network, while the other three gene sets usually surround them, which is consistent with the result shown in Fig. 9a. Their positions within the PPI network clearly show that they are the hub genes in this complicated PPI network. It is expected that hub genes have lower *d*_N_/*d*_S_ values because they are functionally more important than their surrounding neighbors. We further extracted the five most interconnected regions in this network. These regions represent the important sub-networks (modules) within this network. Figure 10b shows the most interconnected module. We can see that *TP53* and *CREBBP* are in the center of this module. They are both POTSFs. In the other modules, POTSFs such as *BRCA1* and *CHEK2* also serve as the hub genes for the sub-networks and some typical oncogenes such as *JUN* and *MYC* are often present in these modules (Figure 13S, 14S, 15S, and 16S).

We also mapped breast cancer, leukemia, and lymphoma drug targets in this PPI network (drug target network is shown in supplemental information, Figure 12S). We found that the drug target genes are scattered in the network instead of congregating in a module or a certain region of the network. The sub-network analyses of drug targets further show that a lot of disease genes are not targeted by any drug (Figure 17S, 18S, and 19S).

## Discussion

Cancer is a disease driven by accumulated somatic mutations which lead to abnormal cell proliferation^9–11^. During the oncogenic transformation process, oncogene and tumor-suppressor genes play contrasting roles in cells. Intriguingly, some genes have both oncogenic and tumor-suppressor functions. In our study, we systematically identified these genes through database search and literature annotation. Our GO analyses show that half of them are transcription factors and can both positively and negatively regulate transcription in cells. Certainly, not all POTSF genes are transcription factors. About one-sixth of them are kinases which catalyze the other protein through phosphorylation. They can increase or decrease a protein’s activity, stabilize or destruct it, and initiate or disrupt its interaction with other proteins^31^. For POTSFs, these contrasting regulatory functions are the functional bases which support their dual roles in cancer development. Furthermore, like ONCs, POTSFs participate in pathways in cancer and chromosomal rearrangement while TSGs do not; like TSGs, POTSFs mainly locate in nucleus while ONCs are enriched in plasma membrane. POTSFs share similarity and dissimilarity with ONCs and TSGs at the same time, which makes them potentially high-value targets for cancer biologists, because selectively tuning their functions is a feasible measure for cancer treatment.

Unsurprisingly, most POTSFs are associated with multiple cancer types. *TP53* alone is implicated in 34 different cancer types. It is worth investigating whether they perform oncogenic or tumor-suppressor function in each associated cancer type through carefully designed experiments, although it is beyond our capability. The proportions of the cancer types associated with POTSFs significantly vary from the proportions of the common cancer types in the 2012 global cancer statistics. Some less common cancer types such as leukemia are over-represented by POTSFs, while some most common cancer types such as lung cancer are under-represented by them. Leukemia, sarcoma, Hodgkin lymphoma, and ovarian cancer are more commonly associated with POTSFs. These cancer types are often epidemically linked to younger populations^32–35^. For example, leukemia is the most common pediatric cancer^36^. Since the number of mutations is positively correlated with age, the younger a cancer patient is, the less mutations his or her cancer has. POTSFs are oncogene and tumor-suppressor gene incorporated into one, and a single mutation event in these genes could theoretically promote its oncogenic function and inhibit its tumor-suppressor function at the same time. Thus, a very few mutations in POTSFs could be sufficient to trigger the cellular cascade of cancerous transformation in vivo.

Our analyses of the non-silent mutation patterns for POTSFs, ONCs, TSGs, and NCRGs show that POTSFs and ONCs accumulate more mutations than TSGs and NCRGs in all 12 cancer types examined in this study. This pattern is also observed in the distribution of potential gain-of-function mutations and potential loss-of-function mutations across 12 cancer types (Figure S4 and S5). One thing we want to mention is that in this study non-silent mutations far outnumber silent mutations by the ratio of 3 to 1 (472,060 vs. 145,294), which propose that these 12 cancers are mainly driven by functionally altering mutations in POTSFs and ONCs. The mutation rate analysis further supported the statement above. In most cancer types examined in this study, POTSFs and/or ONCs have higher mutation rates than TSGs and/or NCTGs. Considering that POTSFs and ONCs averagely have longer gene length than TSGs and NCTGs (Figure S3b), it is unlikely that the mutation bias in POTSFs and ONCs is an artifact of our analysis. Among 12 cancer types, LAML has the lowest number of average non-silent mutations in four gene sets and POTSFs accumulate the highest average number of mutations in this cancer type compared with the other three gene sets. This result is consistent with our observation that some cancer types are specifically associated with POTSFs.

The expression patterns of POTSFs, ONCs, TSGs, and NCRGs are more diversified than their mutation patterns in 12 cancer types. Due to the lack of the expression data from paracancerous tissues, we are unable to confirm whether the expression diversity in 12 cancer types is from their tissue background or the cancer tissue itself. However, through examining their expression level in 6 normal human organs, we find that the down-regulation of TSG expression seem to be the common theme across 12 cancer types. The question is how gain-of-function mutation events could down-regulate the expression of TSGs in cancer cells. One possible scenario is that the gain-of-function mutations in POTSFs promote their oncogenic functions and then the mutated POTSFs negatively regulate TSG expression during cancer development. Interestingly, there is no statistically significant expression difference detected between POTSFs and TSGs in six organs. Moreover, except cerebellum, the median expression level of POTSFs is always higher than that of ONCs. Their expression profile in normal organs suggests that POTSFs function as tumor-suppressor genes rather than oncogenes in normal tissues.

Our network analyses of POTSFs further revealed their possible roles in oncogenesis. Their higher network degree and their position in the PPI network show that POTSFs are more likely to be hub genes in complicated biological networks which have greater influence on cellular functions than non-hub genes. The most interconnected region in our PPI network is centered around *TP53* and *CREBBP* genes. It is no wonder that *TP53* is the POTSF associated with the largest number of cancer types in our study. *TP53* plays an essential role in controlling cell cycle and its mutations have been proved to be associated with many cancer types^37^. Our results again confirmed *TP53* as a super cancer gene from a network perspective. Other well-known cancer genes such as *BRCA1*, *CHEK2*, *JUN*, and *MYC* could also be found in the highly interconnected regions of the PPI network (Figs. 7S, 8S, 9S, and 10S). Actually, like real estate, its position in a network graph can tell a gene’s value in oncogenesis.

Based on next-generation sequencing data, TCGA project has shown that each cancer case has its own unique mutation profile^38^. Thus, the roles of POTSFs in cancer development must vary from case to case. Our study mainly demonstrates that POTSFs are a group of genes with functional duality and prominent PPI network locations. More effort is needed to elucidate their real functions in different cancer types and individual cancer cases, which is also a major research direction in our future study.

## Electronic supplementary material

Supplementary information

Supplemental data

## References

[1] Cancer: the evolutionary legacy. Nat. Med. 6, 496 (2000). 10.1038/74958 10802694

[2] Isobe M, Emanuel BS, Givol D, Oren M, Croce CM (1986). Localization of gene for human p53 tumour antigen to band 17p13. Nature.

[3] Soussi T, Wiman KG (2015). TP53: an oncogene in disguise. Cell Death Differ..

[4] Yang L, Han Y, Suarez Saiz F, Minden MD (2007). A tumor suppressor and oncogene: the WT1 story. Leukemia.

[5] Yip SC, Saha S, Chernoff J (2010). PTP1B: a double agent in metabolism and oncogenesis. Trends Biochem. Sci..

[6] Aster JC, Pear WS, Blacklow SC (2017). The varied roles of Notch in cancer. Annu. Rev. Pathol..

[7] Grabher C, von Boehmer H, Look AT (2006). Notch 1 activation in the molecular pathogenesis of T-cell acute lymphoblastic leukaemia. Nat. Rev. Cancer.

[8] Dotto GP (2009). Crosstalk of Notch with p53 and p63 in cancer growth control. Nat. Rev. Cancer.

[9] Tomasetti C, Vogelstein B (2015). Cancer etiology. Variation in cancer risk among tissues can be explained by the number of stem cell divisions. Science.

[10] Wu S, Powers S, Zhu W, Hannun YA (2016). Substantial contribution of extrinsic risk factors to cancer development. Nature.

[11] Reya T, Morrison SJ, Clarke MF, Weissman IL (2001). Stem cells, cancer, and cancer stem cells. Nature.

[12] Croce CM (2008). Oncogenes and cancer. N. Engl. J. Med..

[13] Oren M, Rotter V (2010). Mutant p53 gain-of-function in cancer. Cold Spring Harb. Perspect. Biol..

[14] Al-Ahmadie HA (2016). Frequent somatic CDH1 loss-of-function mutations in plasmacytoid variant bladder cancer. Nat. Genet..

[15] Belinsky MG (2015). Somatic loss of function mutations in neurofibromin 1 and MYC associated factor X genes identified by exome-wide sequencing in a wild-type GIST case. BMC Cancer.

[16] Weinberg R. A. *The Biology of Cancer*, 2nd edn. (Garland Science, US, 2013).

[17] Knudson AG (2001). Two genetic hits (more or less) to cancer. Nat. Rev. Cancer.

[18] Knudson AG (1971). Mutation and cancer: statistical study of retinoblastoma. Proc. Natl. Acad. Sci. USA.

[19] An O, Dall’Olio GM, Mourikis TP, Ciccarelli FD (2016). NCG 5.0: updates of a manually curated repository of cancer genes and associated properties from cancer mutational screenings. Nucleic Acids Res..

[20] Zhao M, Kim P, Mitra R, Zhao J, Zhao Z (2016). TSGene 2.0: an updated literature-based knowledgebase for tumor suppressor genes. Nucleic Acids Res..

[21] Huang da W, Sherman BT, Lempicki RA (2009). Systematic and integrative analysis of large gene lists using DAVID bioinformatics resources. Nat. Protoc..

[22] Rebhan M, Chalifa-Caspi V, Prilusky J, Lancet D (1997). GeneCards: integrating information about genes, proteins and diseases. Trends Genet..

[23] Chen H, Boutros PC (2011). Venn Diagram: a package for the generation of highly-customizable Venn and Euler diagrams in R. BMC Bioinforma..

[24] Kandoth C (2013). Mutational landscape and significance across 12 major cancer types. Nature.

[25] Cerami E (2012). The cBio cancer genomics portal: an open platform for exploring multidimensional cancer genomics data. Cancer Discov..

[26] Brawand D (2011). The evolution of gene expression levels in mammalian organs. Nature.

[27] Calderone A, Castagnoli L, Cesareni G (2013). mentha: a resource for browsing integrated protein-interaction networks. Nat. Methods.

[28] Shannon P (2003). Cytoscape: a software environment for integrated models of biomolecular interaction networks. Genome Res..

[29] Bader GD, Hogue CW (2003). An automated method for finding molecular complexes in large protein interaction networks. BMC Bioinforma..

[30] Torre LA (2015). Global cancer statistics, 2012. CA Cancer J. Clin..

[31] Manning G, Whyte DB, Martinez R, Hunter T, Sudarsanam S (2002). The protein kinase complement of the human genome. Science.

[32] Smith MA, Altekruse SF, Adamson PC, Reaman GH, Seibel NL (2014). Declining childhood and adolescent cancer mortality. Cancer.

[33] Peppercorn J (2009). Breast cancer in women under 40. Oncol. (Williston. Park)..

[34] Goff BA (2012). Ovarian cancer: screening and early detection. Obstet. Gynecol. Clin. North Am..

[35] Bleyer WA (2002). Cancer in older adolescents and young adults: epidemiology, diagnosis, treatment, survival, and importance of clinical trials. Med. Pediatr. Oncol..

[36] Hutter JJ (2010). Childhood leukemia. Pediatr. Rev..

[37] Martinez E (2015). Comparison of gene expression patterns across 12 tumor types identifies a cancer supercluster characterized by TP53 mutations and cell cycle defects. Oncogene.

[38] Weinstein JN (2013). The Cancer Genome Atlas Pan-Cancer analysis project. Nat. Genet..

