# Supplementary material for: Double agents: genes with both oncogenic and tumor-suppressor functions
Source: Oncogenesis. 2018 Mar 13;7(3):25. doi: 10.1038/s41389-018-0034-x (PMC5852963; doi:10.1038/s41389-018-0034-x)
Supplement: Supplementary file 1 — Supplementary information [file 41389_2018_34_MOESM1_ESM.docx]

Supplementary information for

**Double agents: genes with both oncogenic and tumor-suppressor functions**

Libing Shen^1^, Qili Shi^1^, Wenyuan Wang^1,2*^

Author Affiliations

^1^Interdisciplinary Research Center on Biology and Chemistry, Shanghai Institute of Organic Chemistry, Chinese Academy of Sciences, Shanghai, 200032 P. R. China

^2^Department of Rehabilitation Medicine, Hua Shan Hospital, Fudan University, Shanghai, 200040, P. R. China

^*^Corresponding author: Wenyuan Wang, Email: wywang@sioc.ac.cn

Contents:

1. *The detailed description of gene set gathering process*
2. *Numbers of transcription factors and kinases in POTSFs, ONCs, and TSGs*
3. *Evolutionary origins of POTSFs, ONCs, and TSGs*
4. *Gene property analysis*
5. *Potential gain-of-function mutation, potential loss-of-function mutations, non-silent mutation rates, and silent mutation rates in POTSFs, ONCs, TSGs and NCRGs*
6. *The detailed description of network analysis*
7. *Drug target data*
8. *Module and sub-network figures*
9. *References*
10. *The detailed description of gene set* *gathering process*

Oncogenes (ONCs) were downloaded from Network of Cancer Genes database ([NCG 5.0](https://www.ncbi.nlm.nih.gov/pubmed/26516186)) and tumor-suppressor genes (TSGs) were downloaded from Tumor Suppressor Gene database (TSGene 2.0)[^1^](#_ENREF_1)^,^ [^2^](#_ENREF_2). The genes overlapped between two databases were viewed as the candidate proto-oncogenes with tumor-suppressor function (POTSFs).

First, we searched these candidate genes in the GeneRIF database (ftp://ftp.ncbi.nih.gov/gene/GeneRIF/) which provide the literature annotations for POTSF candidates. Every gene entry in the GeneRIF database is coupled with a PubMed ID which provides a literature evidence for this gene’s biological annotation. Second, the abstract of the literature evidence for each POTSF candidate was retrieved. Then we manually curated these candidate genes according to these abstracts. Only if the literature evidence reported one candidate gene as both oncogene and tumor suppressor gene, it would be identified as proto-oncogene with tumor suppressor function. For example, if one literature reported certain gene as an oncogene in one cancer type while another reported it as a tumor-suppressor gene in another cancer type, it would be regarded as a proto-oncogenes with tumor-suppressor function in our study.

We also reclassified the miscategorized genes (e.g. an oncogene is mistakenly collected as tumor-suppressor gene by one database) and filtered non-protein-coding genes in NCG and TSG databases. The three gene sets gleaned in this step were used for the further analyses.


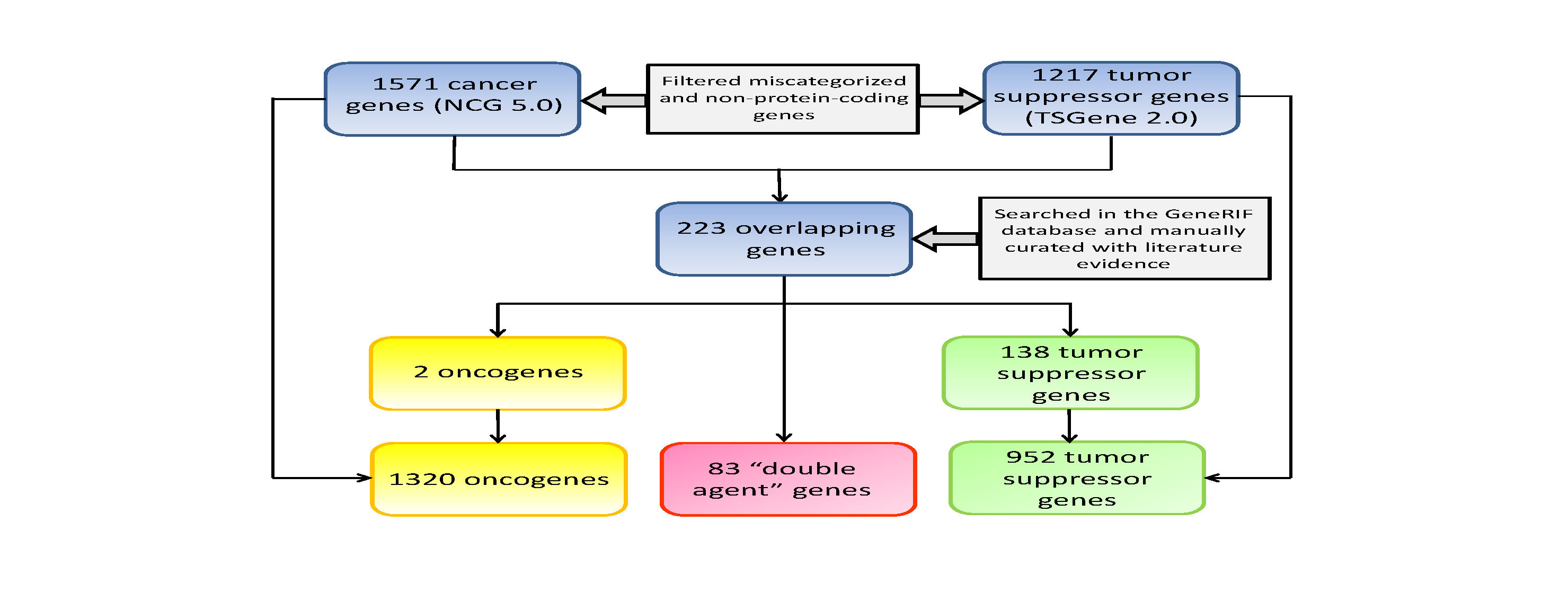


**Figure S1.** Workflow for identifying POTSFs, ONCs, and TSGs.

1. *Numbers of transcription factors and kinases in POTSFs, ONCs, and TSGs*

Because our gene-GO DAVID analysis results showed that the primary molecular process regulated by POTSFs is transcription, we tried to find the percentages of transcription factors and kinases in POTSFs, ONCs, and TSGs. In order to do so, we downloaded the gene names of transcription factors and kinases from PhosphoNetworks and TRRUST databases[^3^](#_ENREF_3)^,^ [^4^](#_ENREF_4). These information was used to calculate the number of transcription factors and kinases in POTSFs, ONCs, and TSGs. If a gene were neither transcription factor nor kinase, it would be viewed as “others” in this study.

We found that about 50% of POTSFs are transcription factors (41/83) and 15.6% of them are kinases (13/83) (Figure S2a). The percentages of transcription factors and kinases are much lower in ONCs and TSGs. 10.6% of ONCs (140/1320) and 12.1% TSGs (115/952) are transcription factors while only 3% of ONCs (39/1320) and 2.3% of TSGs (22/952) are kinases (Figure S2c and S2e).


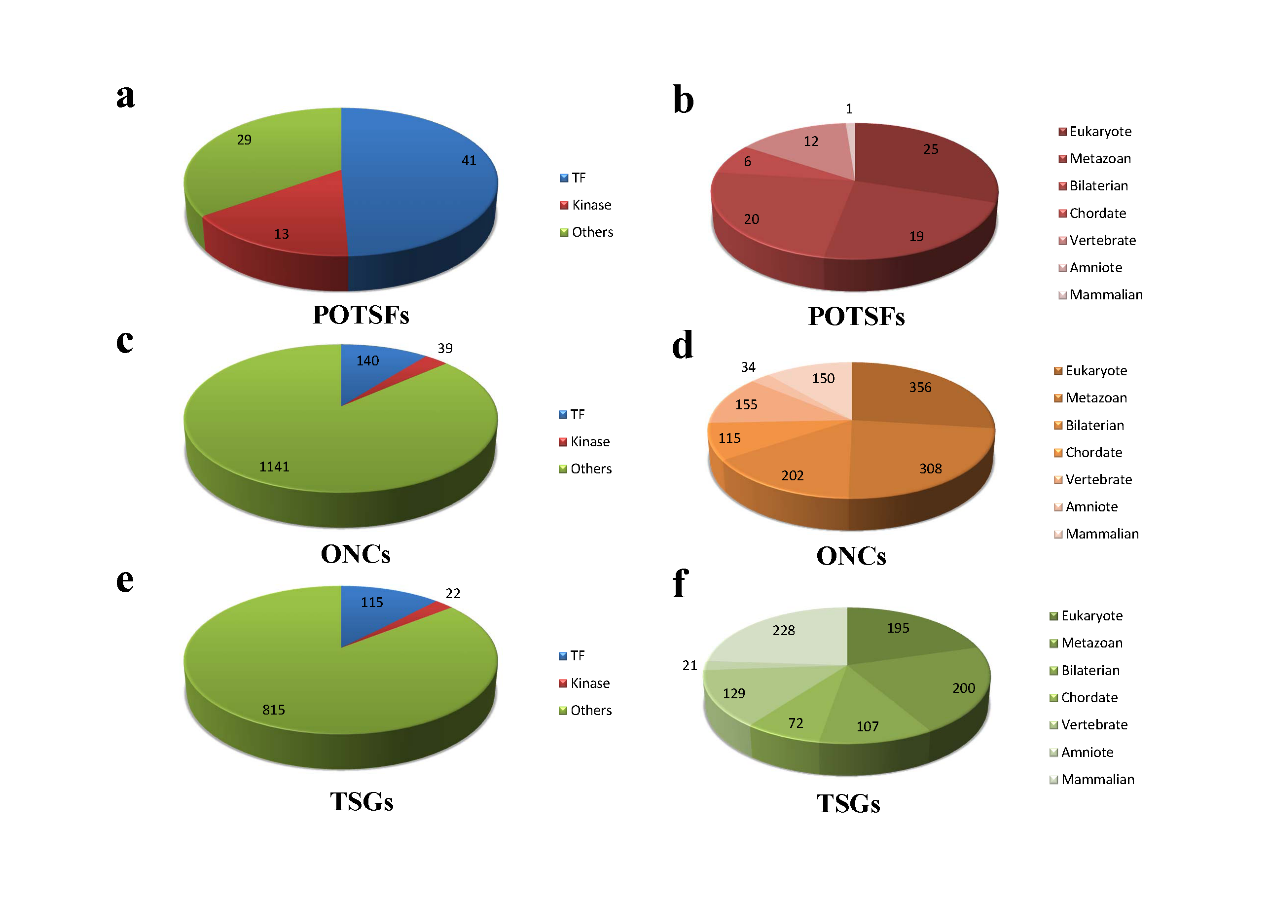


**Figure S2.** Evolutionary origin and gene classification of POTSFs, ONCs, and TSGs. (a) Number of transcription factors and kinases in POTSFs. (b) Evolutionary origins of POTSFs. (c) Number of transcription factors and kinases in ONCs. (d) Evolutionary origins of ONCs. (e) Number of transcription factors and kinases in TSGs. (f) Evolutionary origins of TSGs.

1. *Evolutionary origins of POTSFs, ONCs, and TSGs*

We downloaded the orthologous gene information for ONCs, and TSGs from Ensembl biomart (release 87)[^5^](#_ENREF_5). Ensembl used all-against-all Blast *e*-values (statistical threshold) to cluster genes into orthologous groups[^6^](#_ENREF_6). The species used for orthologous group construction were *Saccharomyces cerevisiae*, *Caenorhabditis elegans*, fruitfly, *C.intestinalis*, zebrafish, and chicken. They represent eukaryote, metazoan, bilaterian, chordate, vertebrate, and amniote, respectively. For an orthologous group, we used the most ancient species to represent their evolutionary origin. If we were unable to find the orthologous representative for a gene among six species above, it would be regarded as originated in mammalian.

We used five representative species to trace the evolutionary origin of each cancer-related gene. The evolutionary origins of POTSFs, ONCs, and TSGs are shown in Figure S2b, S2d, and S2e. 77% of POTSFs, 65% of ONCs, and 52% of TSGs have existed before or since Bilaterian which appeared about 550 million years ago[^7^](#_ENREF_7). This indicates that these genes are greater than 550 million years old. Only 1% of POTSFs belong to the mammalian group, whereas 13% of oncogenes and 30% of tumor-suppressor genes belong to this group. On average, POTSFs are more evolutionarily ancient than ONCs and TSGs. TSGs are the youngest among the three gene sets. This result suggests that POTSFs might perform more fundamental and essential functions for cell survival.

1. *Gene property analysis*

We downloaded the information of gene length, number of transcripts and GC content for all human protein coding genes from Ensembl biomart^[5](#_ENREF_5)^. The genes that are not in POTSFs, ONCs, or TSGs gene set are classified as non-cancer related genes (NCRGs) in this study.

In order to estimate the evolutionary pressure on POTSFs, ONCs, TSGs, and NCRGs, we also downloaded the nonsynonymous and synonymous substitution values between human and mouse genes from Ensembl biomart. Ensembl uses PAML to estimate nonsynonymous and synonymous substitution values between two orthologous genes[^8^](#_ENREF_8). These nonsynonymous and synonymous substitution values were used to calculate the *d*_N_/*d*_S_ ratios for POTSF, ONC, TSG, and NCRGs.

We examined the number of transcripts, gene length, GC content of POTSFs, ONCs, TSGs, and NCRGs. POTSFs have the largest number of transcripts and the longest gene length while NCRGs have the lowest number of transcripts and the shortest gene length (Figure S3a and S3b). Figure 3c shows that POTSFs have the lowest GC content while TSGs have the highest.

Our gene property analyses show that POTSFs have more transcripts and longer gene length than ONCs and TSGs and lower GC-content than TSGs and NCRGs. More transcripts and longer gene length indicate that they are much more versatile in functions and have more and/or longer introns than the other three get sets[^9^](#_ENREF_9)^,^ [^10^](#_ENREF_10), which further proposes that POTSFs are generally more evolutionarily conserved and ancient than ONCs, TSGs, and NCRGs (Figure S2b, S2d and S2f). Collectively, these differences revealed that POTSFs are functionally more diversified and important than ONCs and TSGs.


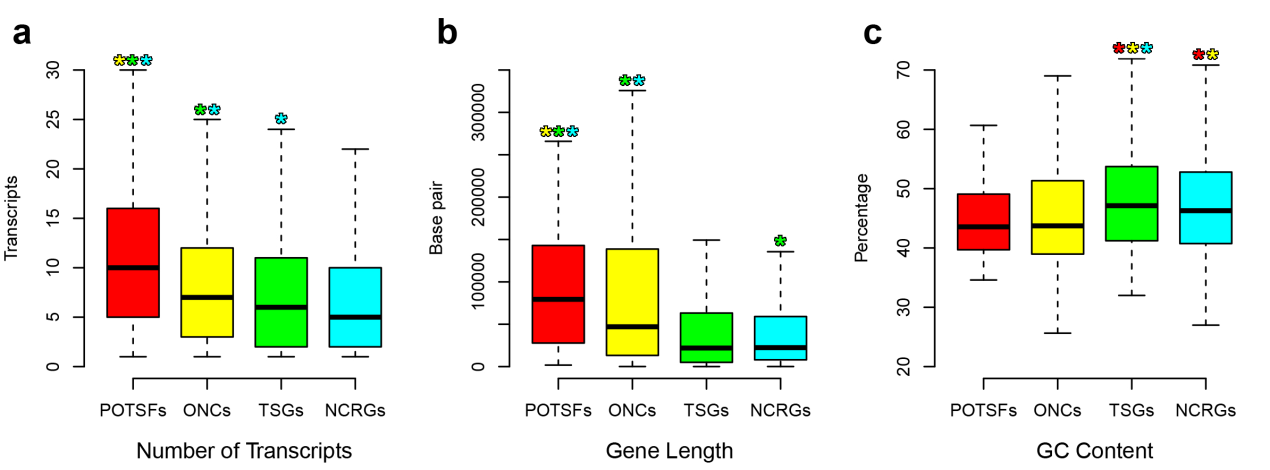


**Figure S3.** Gene and network properties of POTSFs, ONCs, TSGs, and NCRGs. (a) The number of transcripts of four gene sets. (b) The gene length of four gene sets. (c) The GC content of four gene sets.

1. *Potential gain-of-function mutation, potential loss-of-function mutations, non-silent mutation rates and silent mutation rates in POTSFs, ONCs, TSGs and NCRGs*

We further classified the non-silent mutations in this study into two categories --- potential gain-of-function mutations and potential loss-of-function mutations. 416 847 missense mutations and 916 in frame insertions are classified as potential gain-of-function mutations, because they bring different amino acids for a protein. 3 9869 nonsense mutations, 5134 frame shift insertions, 10 743 frame shift deletions, 3 590 in frame deletions, 1 0190 splice site mutations, and 784 nonstop mutations are classified as potential loss-of-function mutations, because they change the reading frame of a coding sequence and potentially result in a malfunctioned protein. Thus, we have total 417 763 potential gain-of-function mutations and total 70 310 potential loss-of-function mutations in this study.

In cancers, gain-of-function mutations are expected to be enriched in oncogenes whereas loss-of-function mutations are expected to be enriched in tumor suppressor genes. Figure S4 shows the distribution of potential gain-of-function mutations in POTSFs, ONCs, TSGs, and NCRGs in all 12 major cancer types. The mutation pattern shown in Figure S4 is almost the same as the distribution pattern of non-silent mutations in in POTSFs, ONCs, TSGs, and NCRGs. It is quite obvious that potential gain-of-function mutations are more concentrated in POTSFs and ONCs than in TSGs and NCRGs. Figure S5 shows the distribution of potential loss-of-function mutations in POTSFs, ONCs, TSGs, and NCRGs in all 12 major cancer types. The mutation pattern shown in Figure S5 is a little bit beyond our expectation, which is nearly the same as the mutation pattern of potential gain-of-function mutations in POTSFs, ONCs, TSGs, and NCRGs. Potential loss-of-function mutations are also biasedly enriched in POTSFs and ONCs instead of TSGs.

We expected that TSGs might have more potential loss-of-function mutations than ONCs, but Figure S5 shows that it is not true at least in the 12 cancer types examined in this study. These results suggest that gain-of-function mutations in POTSFs and ONCs might be more important than loss-of-function mutations in TSGs during the process of cancer development. It is also highly possible that the gain-of-function mutation events in POTSFs abolish their tumor-suppressor function in these 12 cancer types, but experimental confirmation is needed.

Certainly, missense mutation and in frame insertion would reduce protein functionality if occurred at or near the codons for functionally crucial amino acids. Thus, a part of the potential gain-of-function mutations are possibly loss-of-function mutations in our study, especially those in POTSFs. That’s why we labeled missense mutation and in frame insertion as potential gain-of-function mutations, because we are unable to verify their actual effects in each gene.


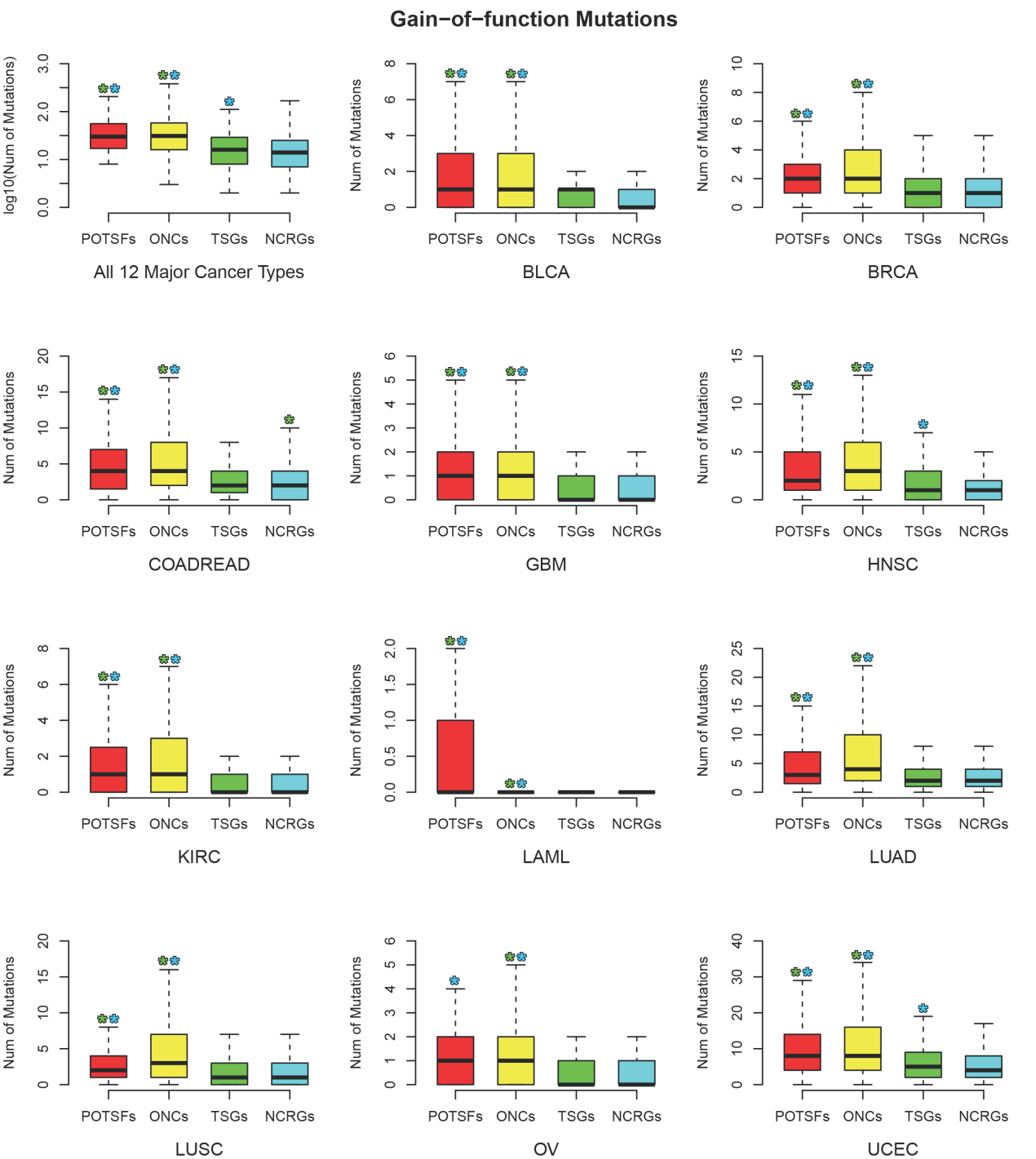


**Figure S4.** The distribution of potential gain-of-function mutations in POTSFs, ONCs, TSGs, and NCRGs across 12 cancer types. The star indicates the statistical difference (*P*-value < 0.05, Kolmogorov–Smirnov test) between two gene sets. The star is placed on the statistically higher gene set and the color of star indicates the corresponding gene set.


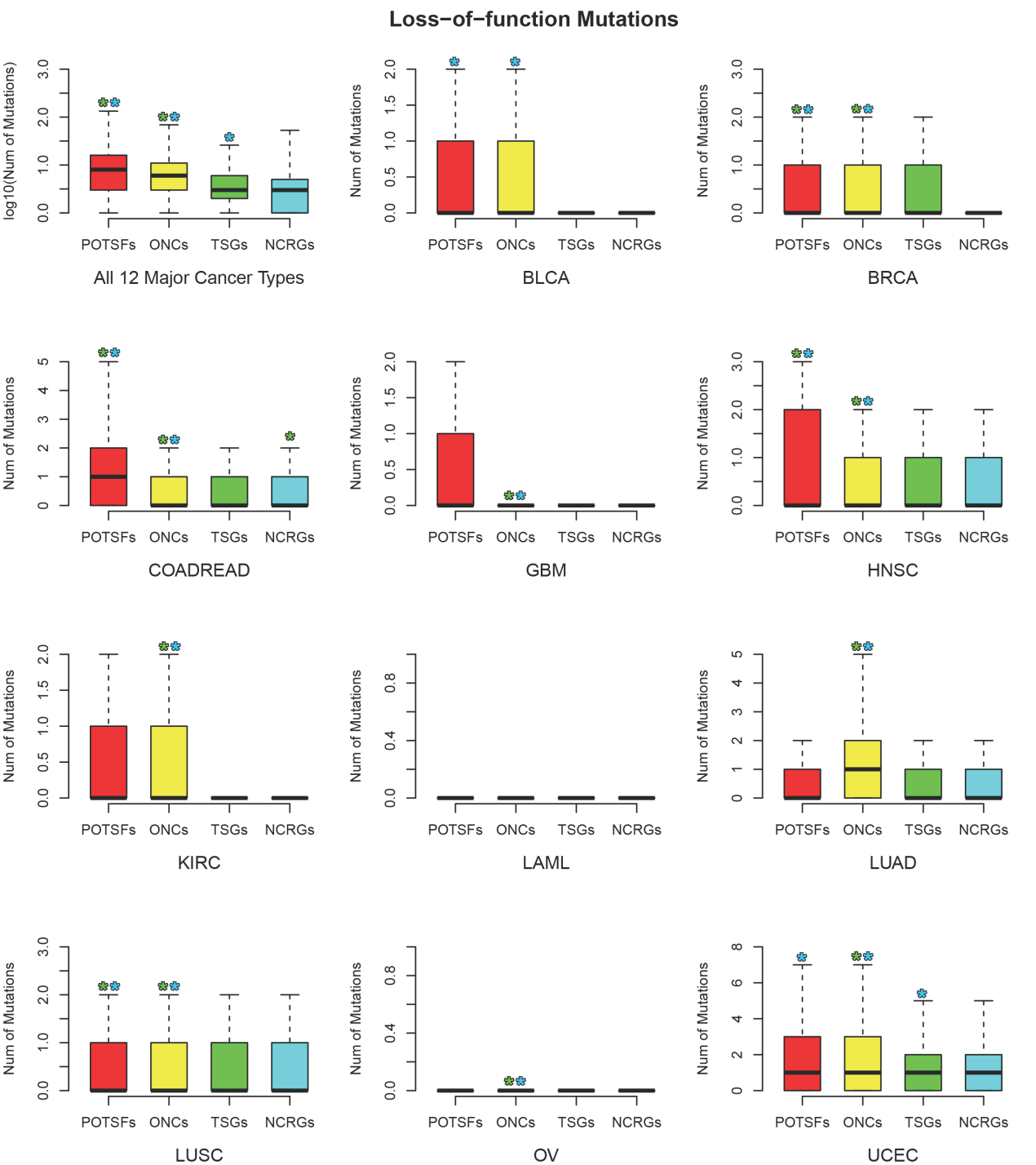


**Figure S5.** The distribution of potential loss-of-function mutations in POTSFs, ONCs, TSGs, and NCRGs across 12 cancer types. The star indicates the statistical difference (*P*-value < 0.05, Kolmogorov–Smirnov test) between two gene sets. The star is placed on the statistically higher gene set and the color of star indicates the corresponding gene set.

We used 472 060 non-silent mutations and 145 294 silent mutations to calculate the non-silent mutation rates and silent mutation rates for POTSFs, ONCs, TSGs and NCRGs in this study. In all 12 cancer types combined, ONCs has the highest non-silent mutation rate among four gene sets, although there is no statistical difference of non-silent mutation rate detected between ONCs and POTSFs. Like non-silent mutation distribution, the non-silent mutation rates of POTSFs, ONCs, TSGs and NCRGs show variations among different cancer types (Figure S6). In BRCA, GBM, HNSC, KIRC, LAML, and OV, POTSFs and ONCs exhibit higher non-silent mutation rates than TSGs and NCRGs. In BLCA and COADREAD, only ONCs exhibit higher non-silent mutation rates than TSGs and NCRGs. In LUAD and LUSC, ONCs has the highest non-silent mutation rate among four gene sets. In UCEC, ONCs and NCRGs have higher non-silent mutation rates than POTSFs and TSGs. In all 12 cancer types combined, the silent mutation rates of POTSFs, ONCs, TSGs and NCRGs show a quite different pattern from their non-silent mutation rates (the first panel of Figure S7). However, the silent mutation rates of POTSFs, ONCs, TSGs and NCRGs are very similar to their non-silent mutation rates in each individual cancer type (the other panels of Figure S7). We found that that the exceptionally high silent mutation rate in UCEC led to the discrepancy between non-silent mutation rate and silent mutation rate among four gene sets in all 12 cancer types combined.


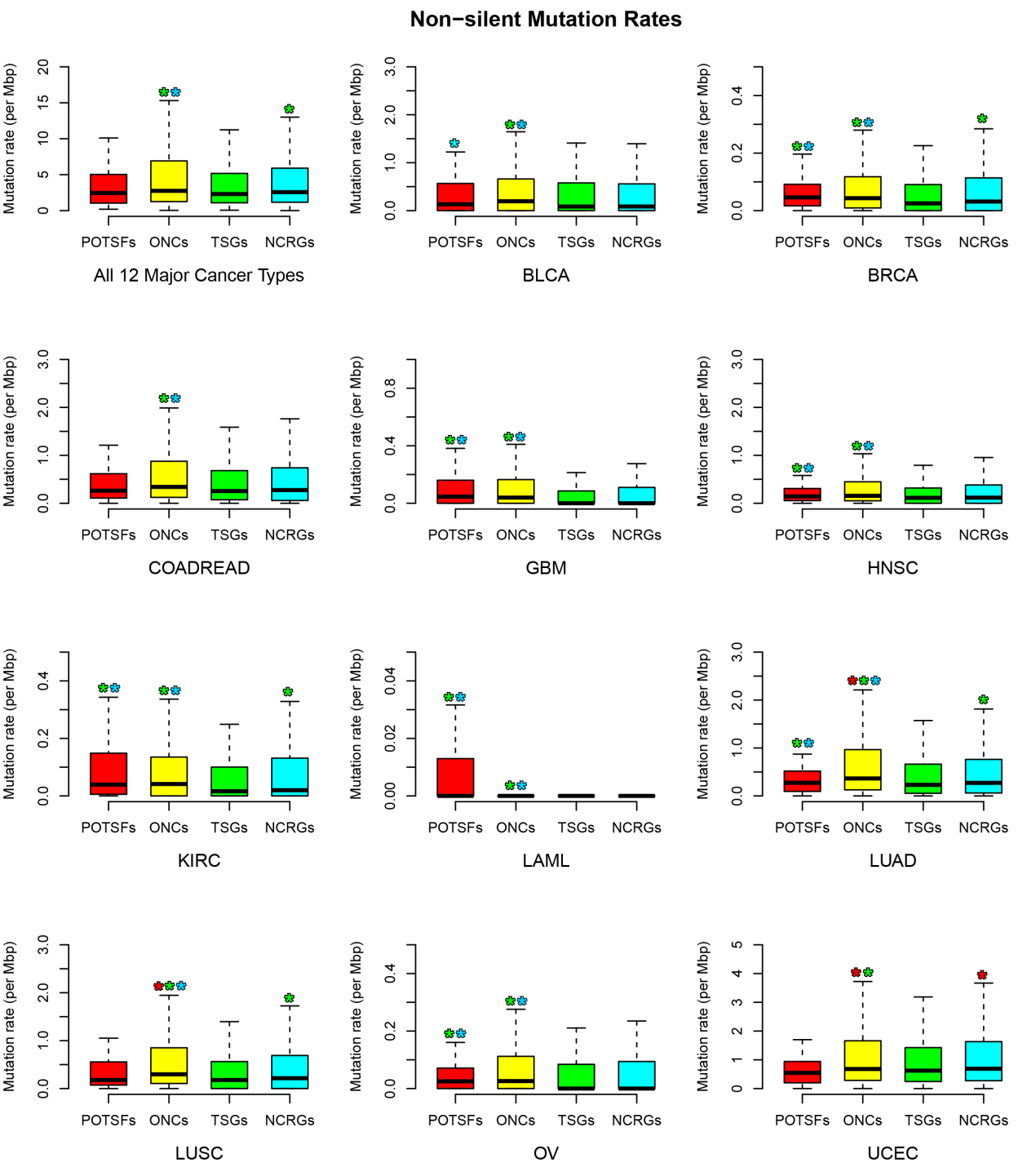


**Figure S6.** The non-silent mutation rates in POTSFs, ONCs, TSGs, and NCRGs across 12 cancer types. The star indicates the statistical difference (*P*-value < 0.05, Kolmogorov–Smirnov test) between two gene sets. The star is placed on the statistically higher gene set and the color of star indicates the corresponding gene set.


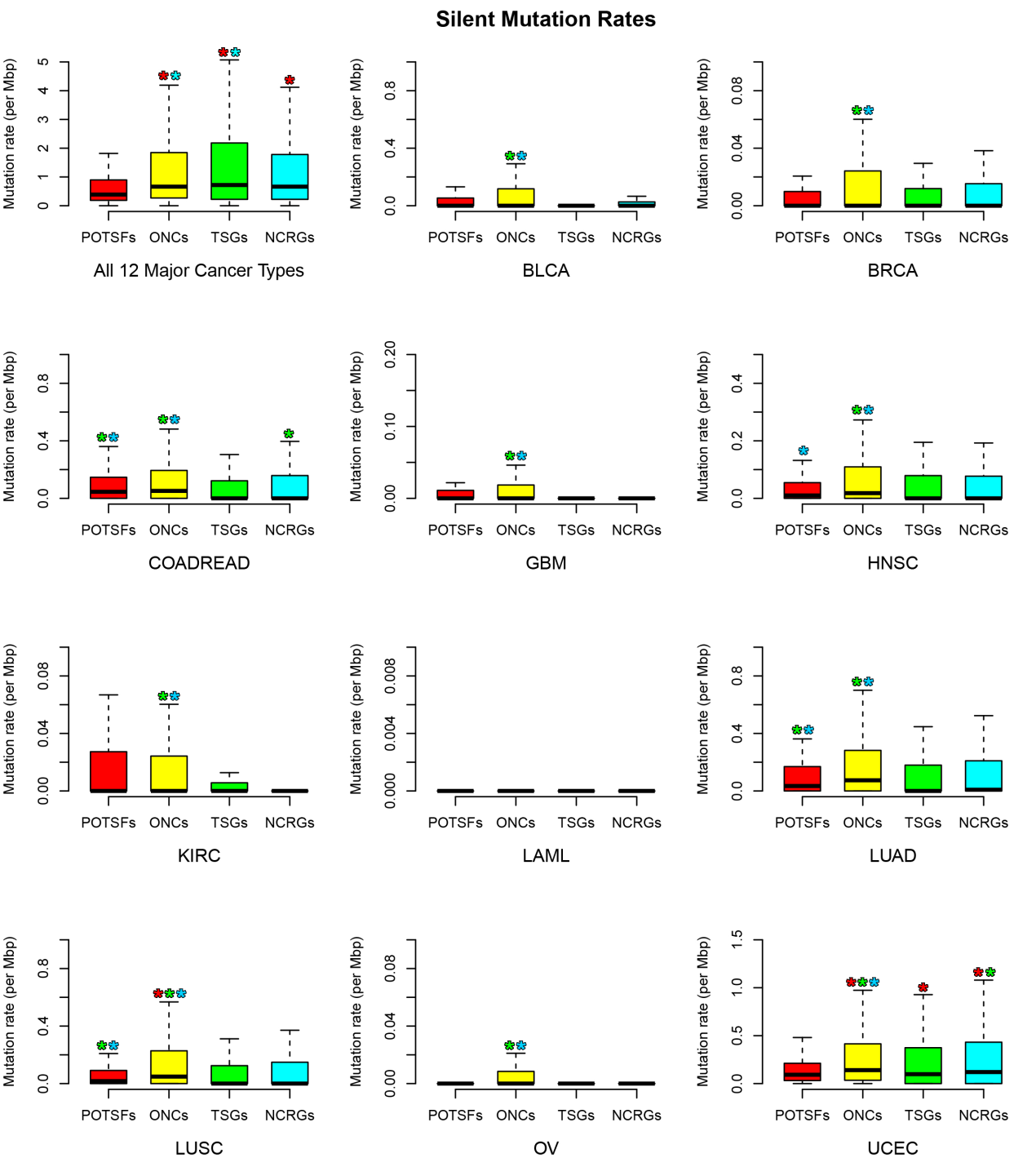


**Figure S7.** The silent mutation rates in POTSFs, ONCs, TSGs, and NCRGs across 12 cancer types. The star indicates the statistical difference (*P*-value < 0.05, Kolmogorov–Smirnov test) between two gene sets. The star is placed on the statistically higher gene set and the color of star indicates the corresponding gene set.

1. *The detailed description of network analysis*

We downloaded the protein-protein interaction information from mentha database and used them to perform network analysis in this study[^11^](#_ENREF_11).

After filtered the non-human proteins in mentha dataset, we obtained the interaction information for 17 644 human proteins which include 83 POTSFs, 1 139 ONCs, 770 TSGs, and 15 652 NCRGs.

For each protein, we calculated its degree *k* and clustering coefficient *C*(*k*). Degree is a measure of a node’s connectivity in a network. In protein-interaction network, it measures how many interaction neighbors (neighbor proteins) a protein has. Clustering coefficient measures a node’s modularity in a network, i.e. the degree to which nodes in a network tend to cluster together. In protein-protein interaction network, modularity implies certain biological function.

The clustering coefficient is mathematically defined as follows[^12^](#_ENREF_12):

$$C\left( k \right)=\frac{2n}{k\left( k-1 \right)}$$

where *n* is the number of direct links among a specific gene’s neighbors and *k*(*k* − 1)/2 is the total possible number of direct links among its neighbors.

Biological networks can be characterized by the power-law degree distribution and hierarchical structure[^12^](#_ENREF_12). In this study, we also examined the network characteristics of POTSFs, ONCs, TSGs, and NCRGs. Figure S8a shows that the degree distribution of POTSFs to the probability *P(k)* doesn’t fit the power law (the power-law fit is shown as a red line, *R^2^* = 0.03, *P*-value = 0.056). Figure S9a, S10a, and S11a show that the degree distribution of ONCs, TSGs, or NCRGs to the probability *P(k)* fit the power law (*R^2^* > 0.8, *P*-values < 2×10^−16^). Figure S8b, S9b, S10b and S11b show that the scaling of the clustering coefficient of OTSFs, ONCs, TSGs, or NCRGs to log(*K*) follows *C(K)*~*K^−1^* (which is shown in a red straight line with negative slope, *P*-values < 2×10^−16^).

That a gene set’s degree distribution fits the power law indicates that they constitute a random network. Our result proposes that POTSFs don’t constitute a random network, which is either due to the small number of POTSFs in this study or due to most of them with a large number of network neighbors. Nevertheless, the scaling of the clustering coefficient of POTSFs, ONCs, TSGs, or NCRGs shows that all four gene sets have a hierarchical network structure (sparsely connected nodes are part of highly clustered areas and different clustered areas are communicated by a few hubs).


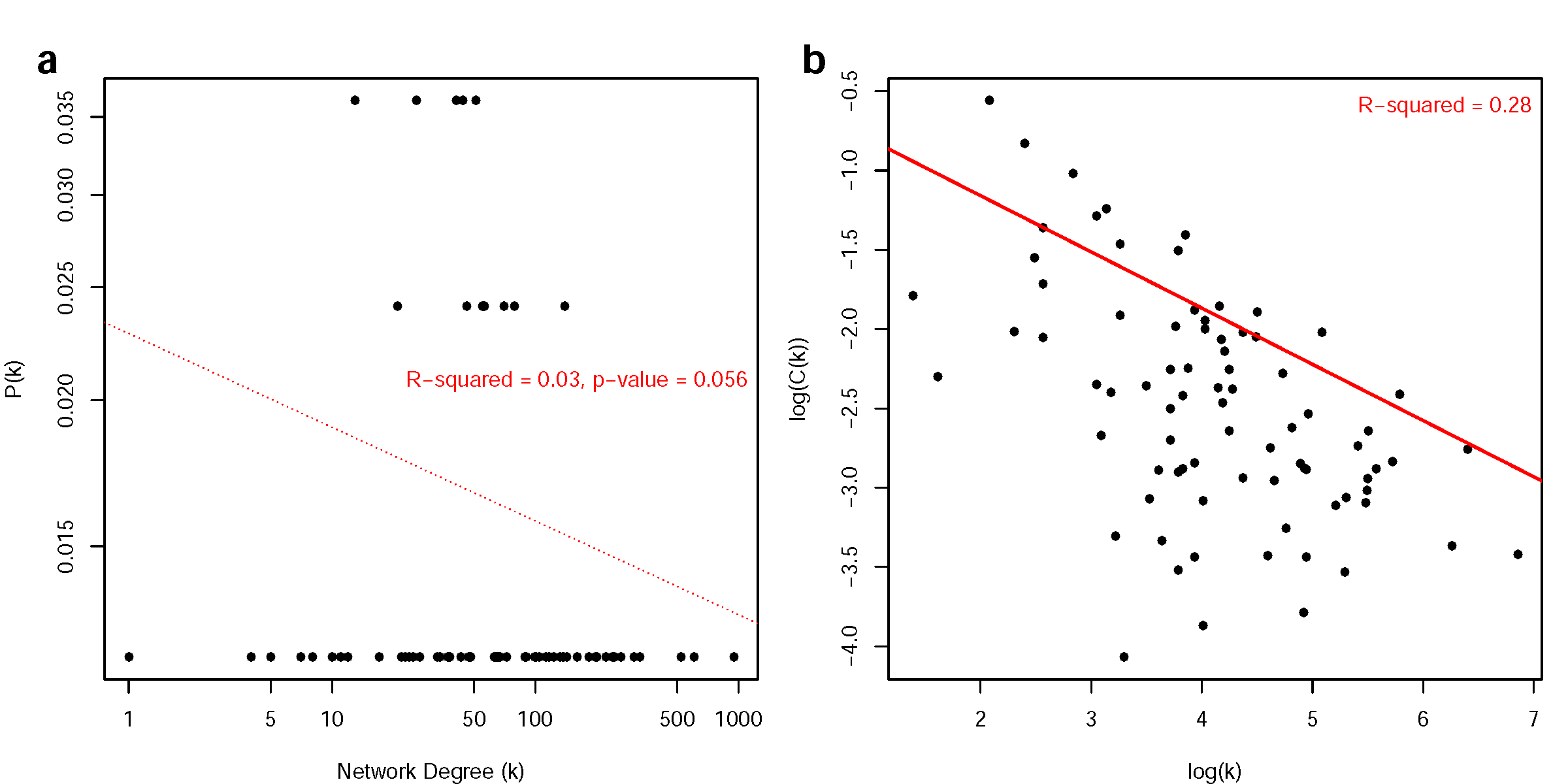


**Figure S8.** Network characteristics of POTSFs. (a) Degree Distribution of POTSFs. (b) Scaling of the clustering coefficient in POTSFs.


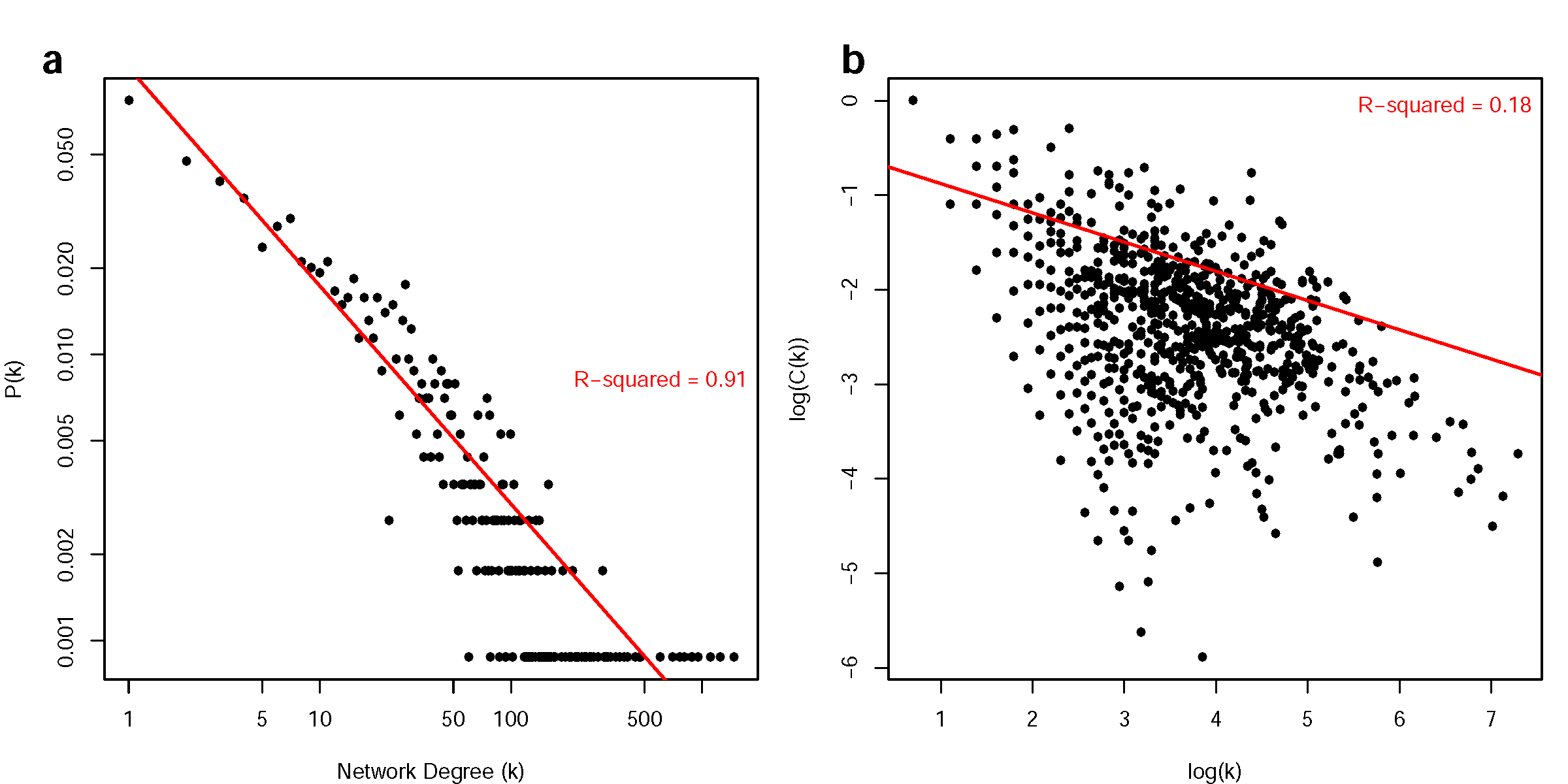


**Figure S9.** Network characteristics of ONCs. (a) Degree Distribution of ONCs. (b) Scaling of the clustering coefficient in ONCs.


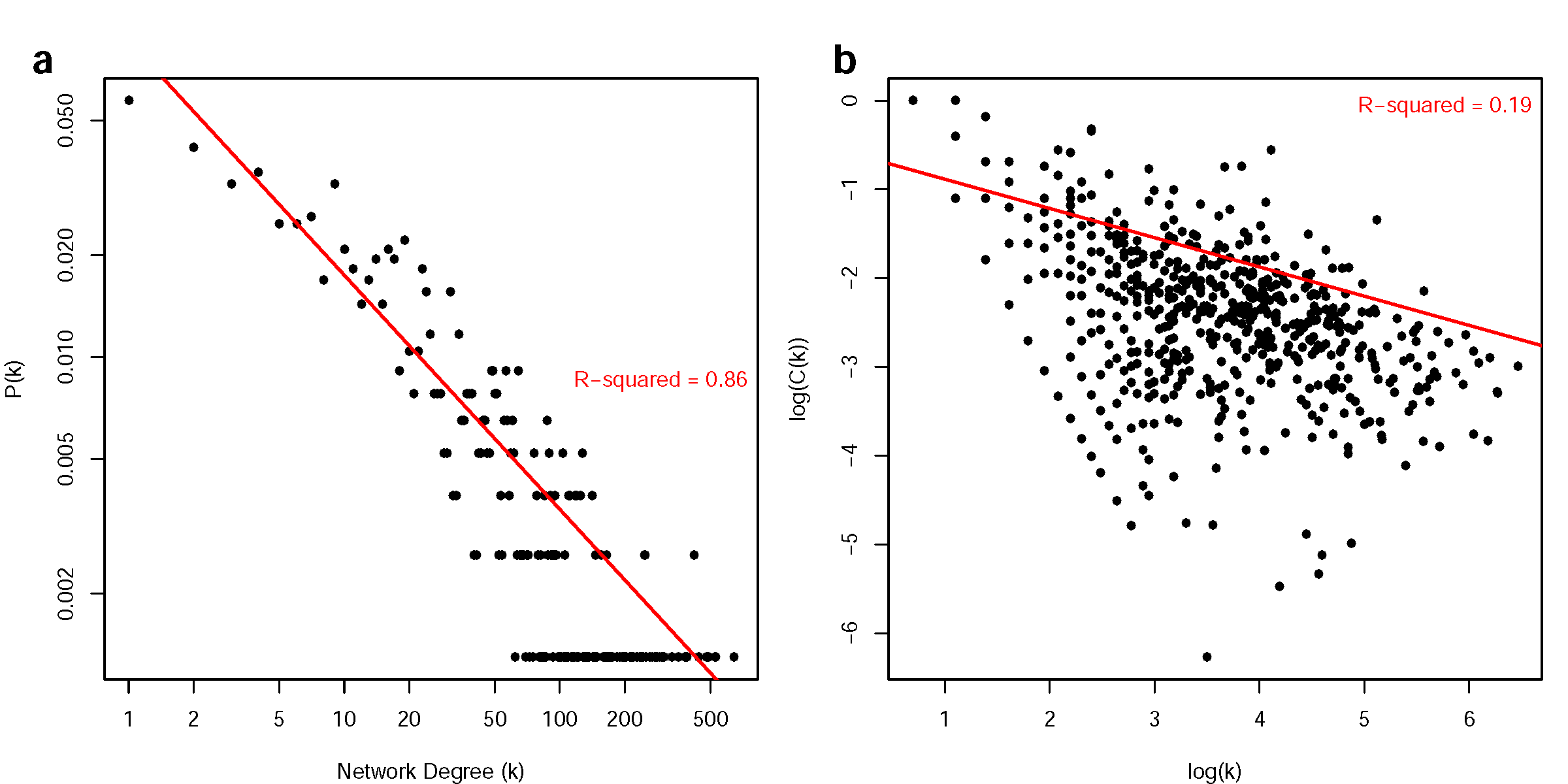


**Figure S10.** Network characteristics of TSGs. (a) Degree Distribution of TSGs. (b) Scaling of the clustering coefficient in TSGs.


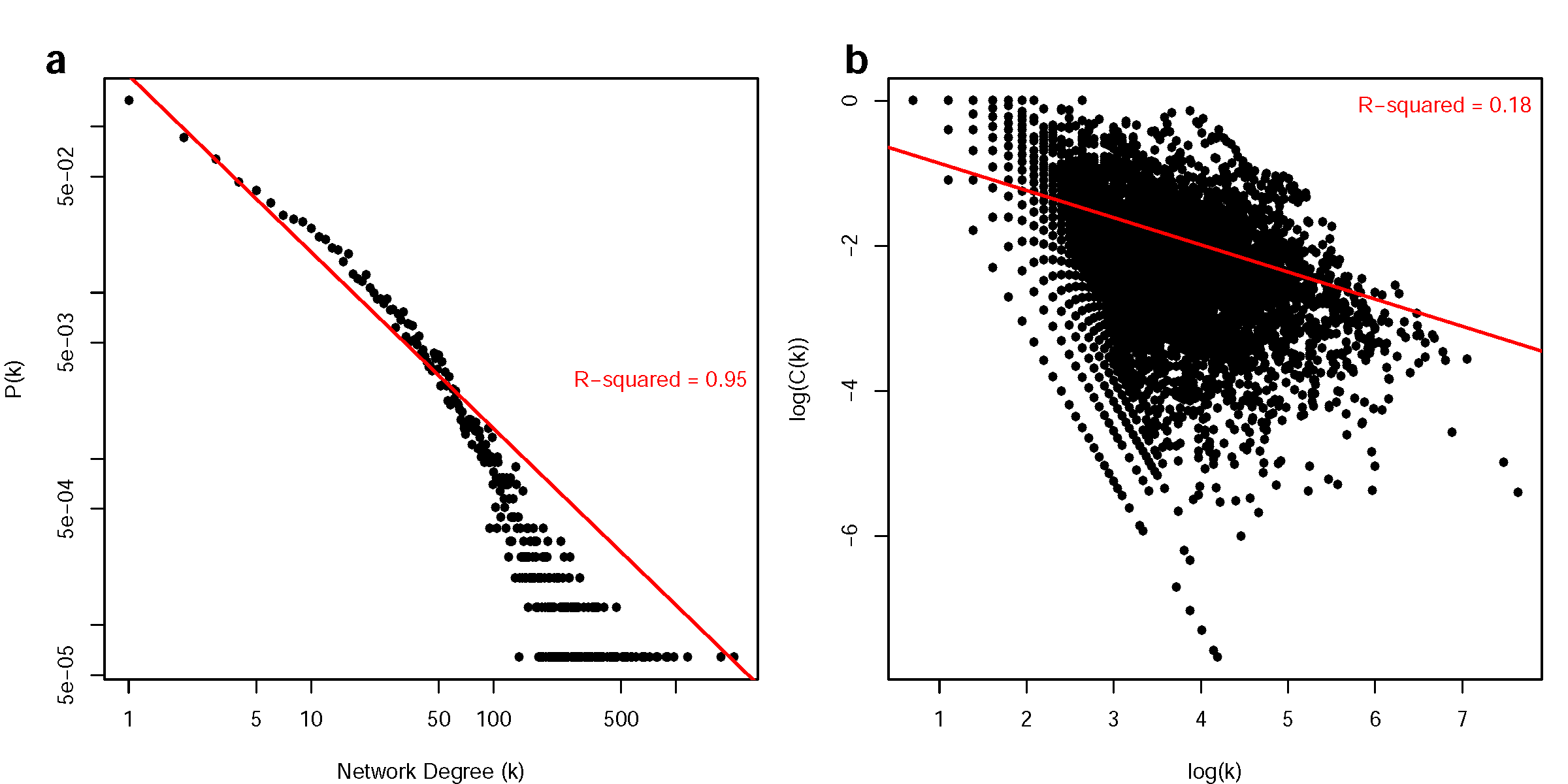


**Figure S11.** Network characteristics of NCRGs. (a) Degree Distribution of NCRGs. (b) Scaling of the clustering coefficient in NCRGs.

1. *Drug target data*

The drug target data were downloaded from the supplementary information of Yıldırım et al[^13^](#_ENREF_13). We extracted the drug target genes in breast cancer, leukemia and lymphoma and their related drugs from the downloaded table. There are 22 drugs and 19 drug target genes in breast cancer, 31 drugs and 37 drug target genes in leukemia, and 17 drugs and 10 drug target genes in lymphoma, respectively.

We mapped the drug target genes in protein-protein interaction network (Figure S12). We also generated the highly interconnected sub-network for drug target genes and their interaction genes based on protein-protein interaction information. Because all drugs target the same group of genes in one cancer type, we used one drug as the representative in the sub-network. The representative drugs for treating breast cancer, leukemia, and lymphoma are Capecitabine, Prednisolone, and Bexarotene, respectively. We collected the disease information for the genes in sub-networks through literature-search and text-mining (ftp://ftp.ncbi.nih.gov/gene/GeneRIF/).


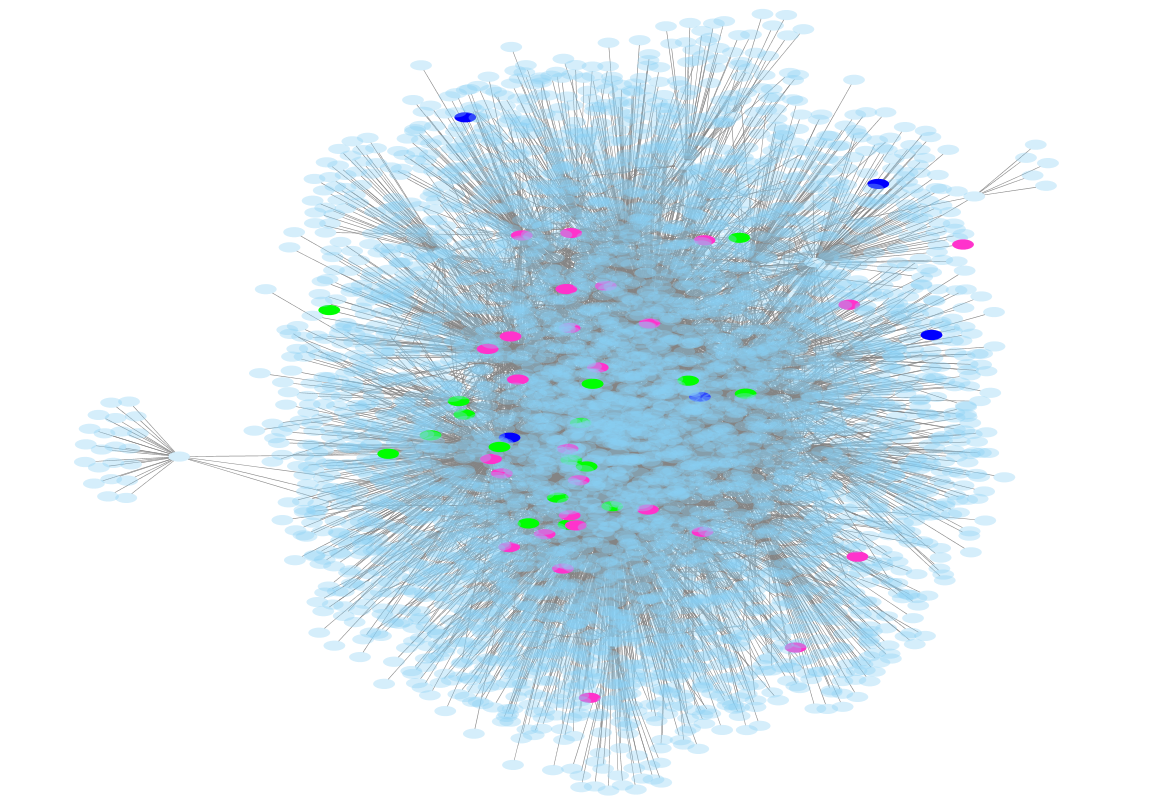


**Figure S12.** Distribution of drug target genes in protein-protein interaction network. Green indicates breast cancer drug targets. Magenta indicates leukemia drug targets. Dark blue indicates lymphoma drug targets.

1. *Module and sub-network figures*


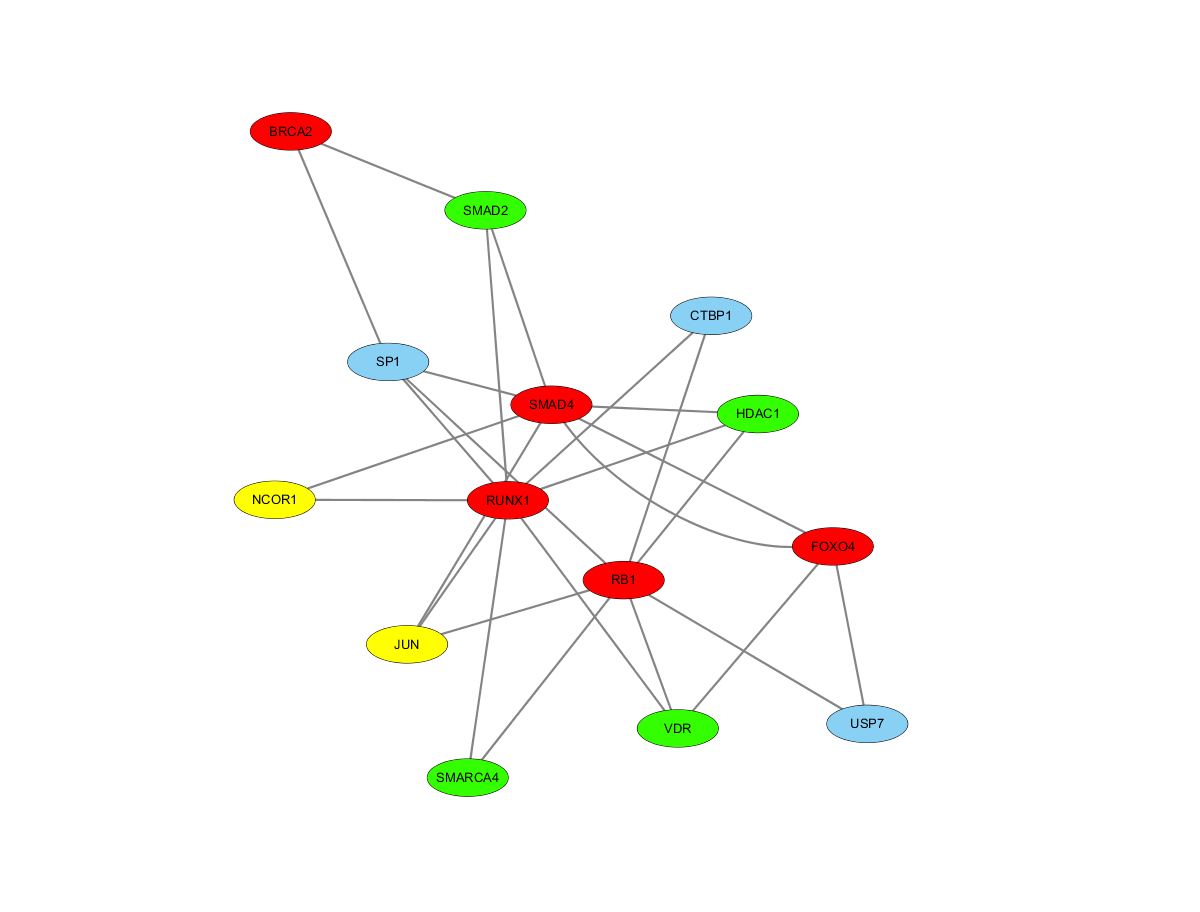


**Figure S13.** The second most interconnected region of the PPI network. POTSFs are represented by red color. ONCs are represented by yellow color. TSGs are represented by green color. NCRGs are represented by blue color.


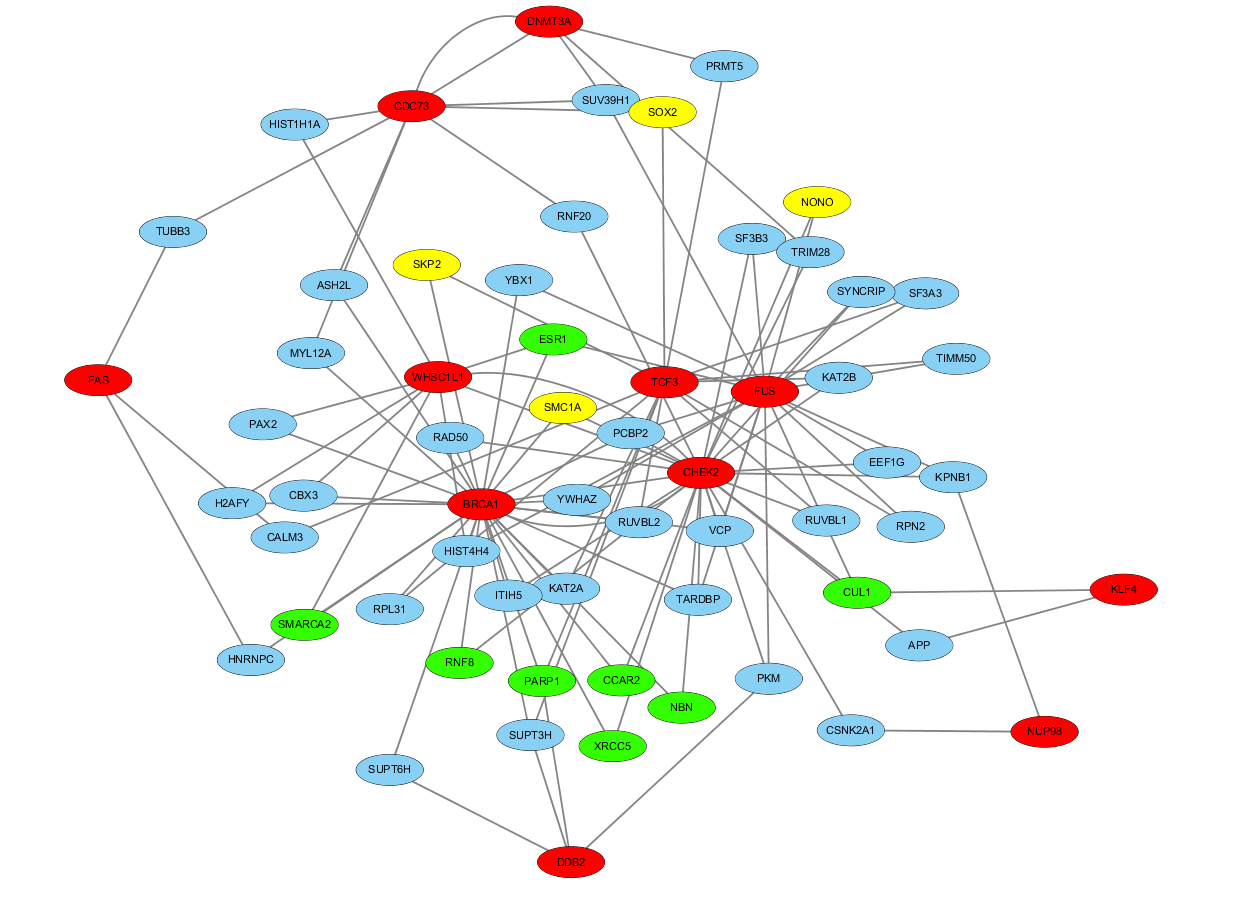


**Figure S14.** The third most interconnected region of the PPI network. POTSFs are represented by red color. ONCs are represented by yellow color. TSGs are represented by green color. NCRGs are represented by blue color.


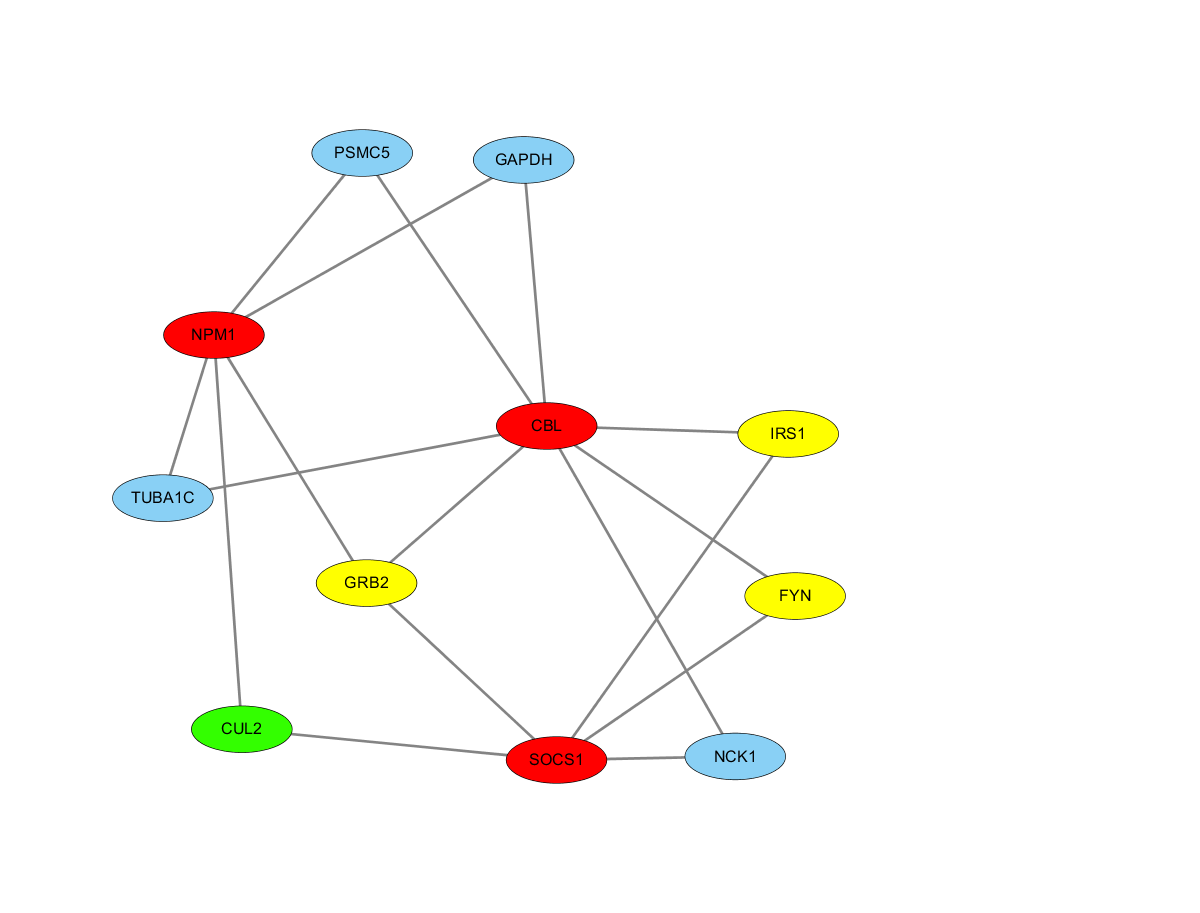


**Figure S15.** The fourth most interconnected region of the PPI network. POTSFs are represented by red color. ONCs are represented by yellow color. TSGs are represented by green color. NCRGs are represented by blue color.


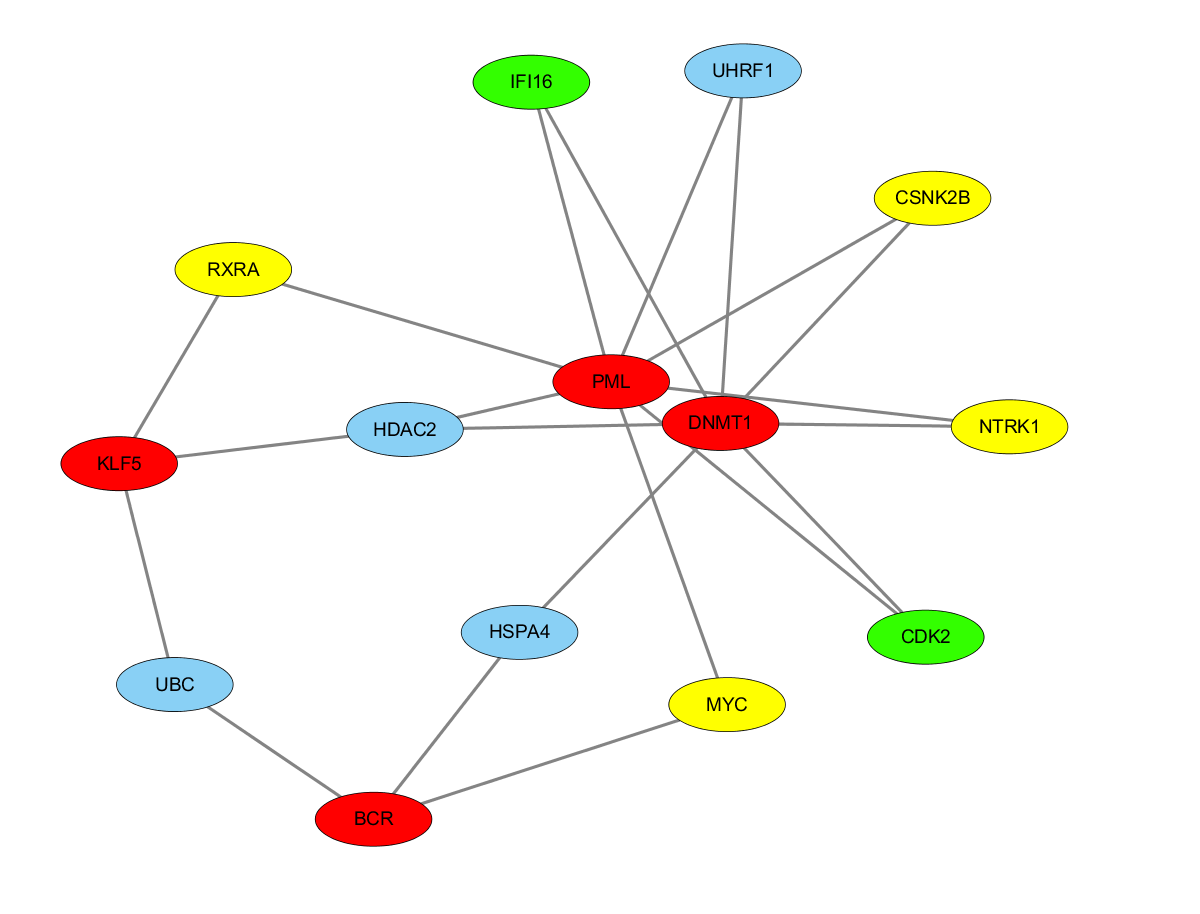


**Figure S16.** The fifth most interconnected region of the PPI network. POTSFs are represented by red color. ONCs are represented by yellow color. TSGs are represented by green color. NCRGs are represented by blue color.


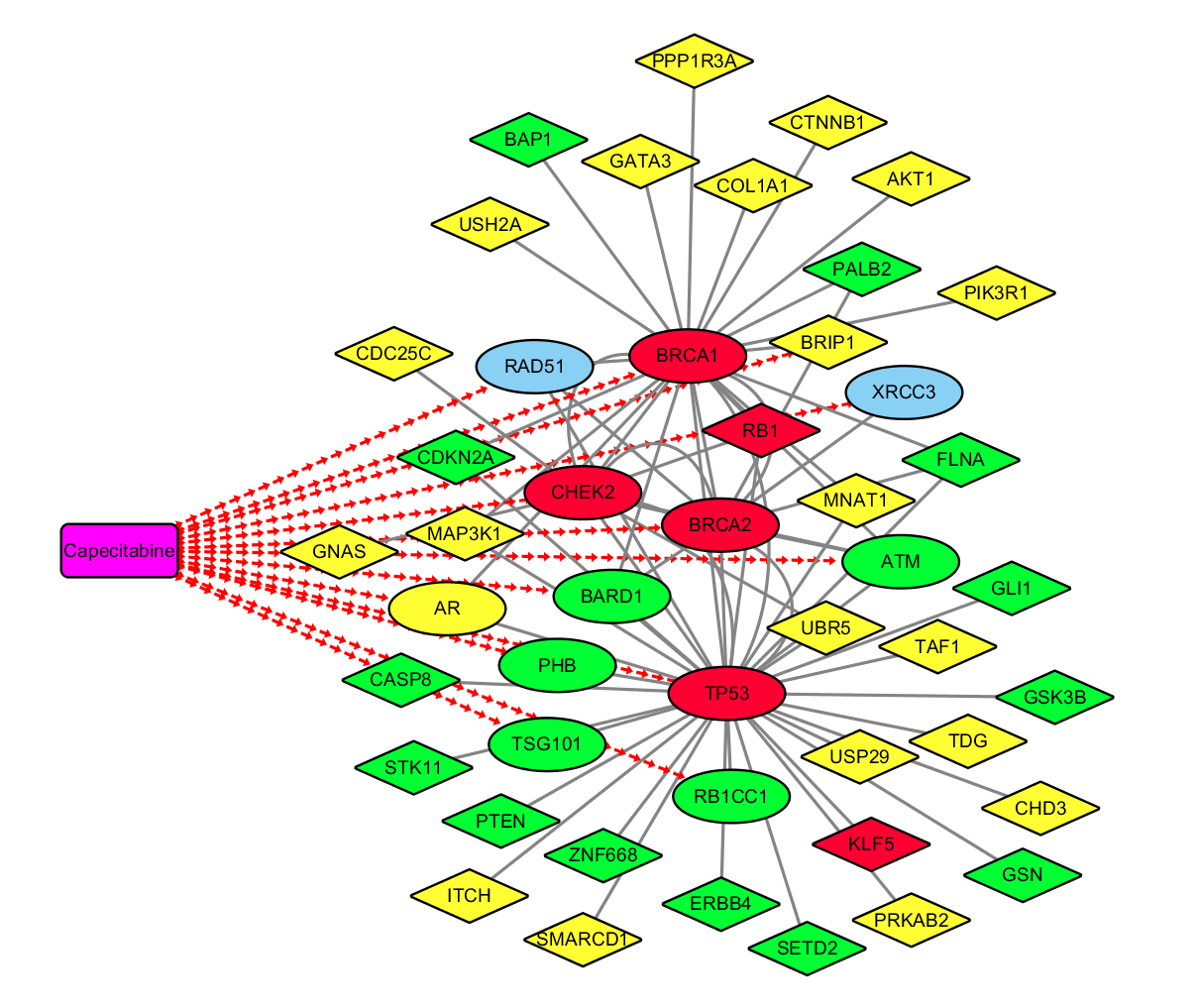


**Figure S17.** The sub-network for breast cancer drug targets. POTSFs are represented by red color. ONCs are represented by yellow color. TSGs are represented by green color. NCRGs are represented by blue color. Drug is represented by Magenta. ○ Ellipse indicates breast cancer drug target genes. ◊ Rhombus indicates the genes associated with breast cancer but not targeted by any drug.


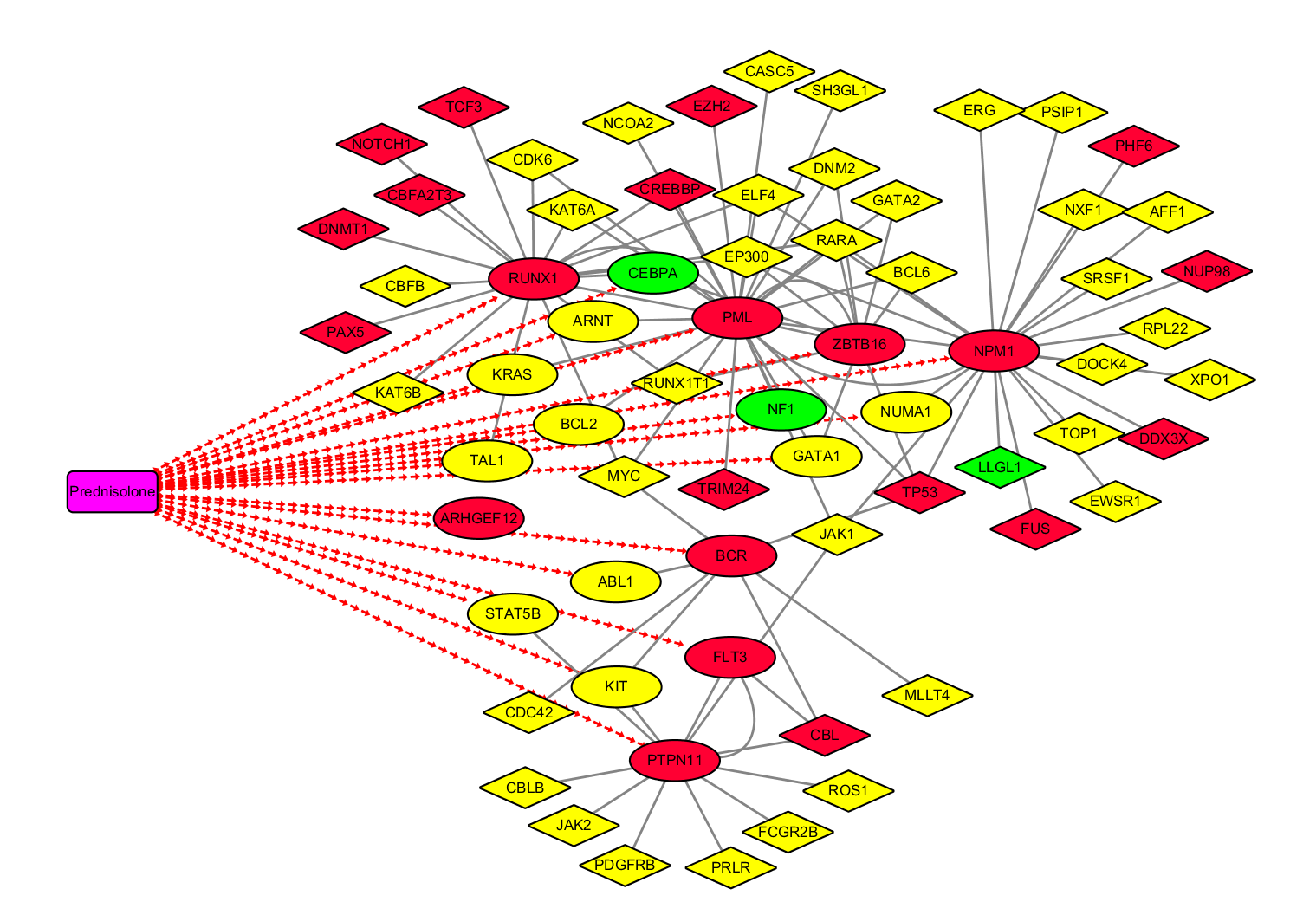


**Figure S18.** The sub-network for leukemia drug targets. POTSFs are represented by red color. ONCs are represented by yellow color. TSGs are represented by green color. NCRGs are represented by blue color. Drug is represented by Magenta. ○ Ellipse indicates leukemia drug target genes. ◊ Rhombus indicates the genes associated with leukemia but not targeted by any drug.


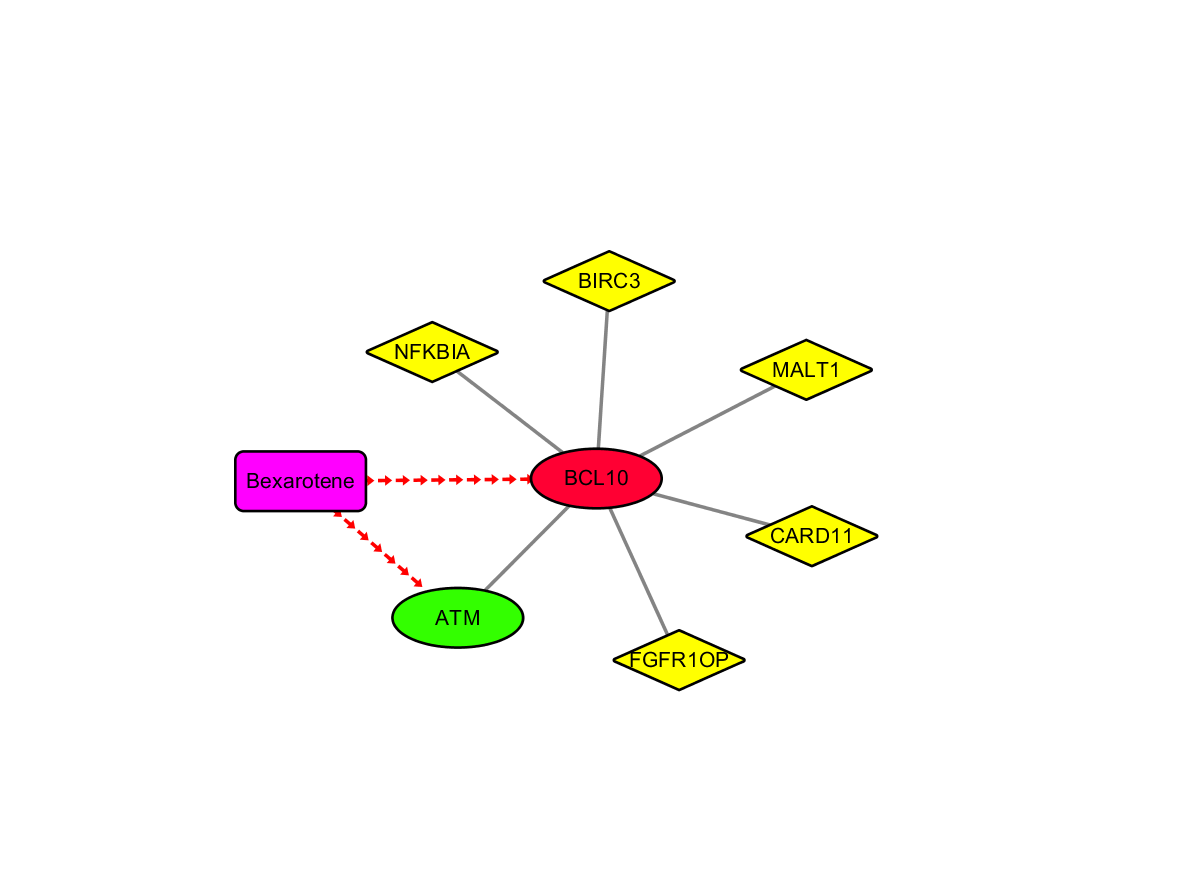


**Figure S19.** The sub-network for lymphoma drug targets. POTSFs are represented by red color. ONCs are represented by yellow color. TSGs are represented by green color. NCRGs are represented by blue color. Drug is represented by Magenta. ○ Ellipse indicates lymphoma drug target genes. ◊ Rhombus indicates the genes associated with lymphoma but not targeted by any drug.

1. *References*

1 An O, Dall'Olio GM, Mourikis TP, Ciccarelli FD. NCG 5.0: updates of a manually curated repository of cancer genes and associated properties from cancer mutational screenings. Nucleic acids research 2016; 44: D992-999.

2 Zhao M, Kim P, Mitra R, Zhao J, Zhao Z. TSGene 2.0: an updated literature-based knowledgebase for tumor suppressor genes. Nucleic acids research 2016; 44: D1023-1031.

3 Hu J, Rho HS, Newman RH, Zhang J, Zhu H, Qian J. PhosphoNetworks: a database for human phosphorylation networks. Bioinformatics 2014; 30: 141-142.

4 Han H, Shim H, Shin D, Shim JE, Ko Y, Shin J *et al*. TRRUST: a reference database of human transcriptional regulatory interactions. Scientific reports 2015; 5: 11432.

5 Kinsella RJ, Kahari A, Haider S, Zamora J, Proctor G, Spudich G *et al*. Ensembl BioMarts: a hub for data retrieval across taxonomic space. Database : the journal of biological databases and curation 2011; 2011: bar030.

6 Slater GS, Birney E. Automated generation of heuristics for biological sequence comparison. BMC bioinformatics 2005; 6: 31.

7 Knoll AH, Carroll SB. Early animal evolution: emerging views from comparative biology and geology. Science 1999; 284: 2129-2137.

8 Herrero J, Muffato M, Beal K, Fitzgerald S, Gordon L, Pignatelli M *et al*. Ensembl comparative genomics resources. Database : the journal of biological databases and curation 2016; 2016.

9 Gingeras TR. Origin of phenotypes: genes and transcripts. Genome research 2007; 17: 682-690.

10 Roy SW, Penny D. Intron length distributions and gene prediction. Nucleic acids research 2007; 35: 4737-4742.

11 Calderone A, Castagnoli L, Cesareni G. mentha: a resource for browsing integrated protein-interaction networks. Nature methods 2013; 10: 690-691.

12 Barabasi AL, Oltvai ZN. Network biology: understanding the cell's functional organization. Nature reviews Genetics 2004; 5: 101-113.

13 Yildirim MA, Goh KI, Cusick ME, Barabasi AL, Vidal M. Drug-target network. Nature biotechnology 2007; 25: 1119-1126.
